# The Gβ-like protein Bcgbl1 regulates development and pathogenicity of the gray mold *Botrytis cinerea via* modulating two MAP kinase signaling pathways

**DOI:** 10.1371/journal.ppat.1011839

**Published:** 2023-12-04

**Authors:** Jiejing Tang, Zhe Sui, Ronghui Li, Yuping Xu, Lixuan Xiang, Shiying Fu, Jinfeng Wei, Xuan Cai, Mingde Wu, Jing Zhang, Weidong Chen, Yangdou Wei, Guoqing Li, Long Yang

**Affiliations:** 1 State Key Laboratory of Agricultural Microbiology, Huazhong Agricultural University, Wuhan, China; 2 Hubei Key Laboratory of Plant Pathology, College of Plant Science and Technology, Huazhong Agricultural University, Wuhan, China; 3 U.S. Department of Agriculture, Agricultural Research Service, Washington State University, Pullman, Washington, United States of America; 4 Department of Biology, University of Saskatchewan, Saskatoon, Saskatchewan, Canada; Purdue University, UNITED STATES

## Abstract

The fungal Gβ-like protein has been reported to be involved in a variety of biological processes, such as mycelial growth, differentiation, conidiation, stress responses and infection. However, molecular mechanisms of the Gβ-like protein in regulating fungal development and pathogenicity are largely unknown. Here, we show that the Gβ-like protein gene *Bcgbl1* in the gray mold fungus *Botrytis cinerea* plays a pivotal role in development and pathogenicity by regulating the mitogen-activated protein (MAP) kinases signaling pathways. The *Bcgbl1* deletion mutants were defective in mycelial growth, sclerotial formation, conidiation, macroconidial morphogenesis, plant adhesion, and formation of infection cushions and appressorium-like structures, resulting in a complete loss of pathogenicity. Bcgbl1 interacted with BcSte50, the adapter protein of the cascade of MAP kinase (MAPK). *Bcgbl1* mutants had reduced phosphorylation levels of two MAPKs, namely Bmp1 and Bmp3, thereby reducing infection. However, deletion of *Bcgbl1* did not affect the intracellular cAMP level, and exogenous cAMP could not restore the defects. Moreover, *Bcgbl1* mutants exhibited defects in cell wall integrity and oxidative stress tolerance. Transcriptional profiling revealed that Bcgbl1 plays a global role in regulation of gene expression upon hydrophobic surface induction. We further uncovered that three target genes encoding the hydrophobic surface binding proteins (HsbAs) contributed to the adhesion and virulence of *B*. *cinerea*. Overall, these findings suggest that Bcgbl1 had multiple functions and provided new insights for deciphering the Bcgbl1-mediated network for regulating development and pathogenicity of *B*. *cinerea*.

## Author summary

*Botrytis cinerea* is an important phytopathogenic fungus that causes severe diseases in over 1,400 plant species worldwide. It has been known that the Gβ-like protein plays an important role in fungal development and virulence. However, the regulatory network mediated by the Gβ-like protein and its downstream targets remain elusive. Here, we report that the Gβ-like protein Bcgbl1 is essential for development and pathogenicity of *B*. *cinerea*. We showed that the Bcgbl1 protein physically interacted with the MAPK adaptor protein Ste50 and activated two distinct MAPKs (Bmp1 and Bmp3) that regulate fungal adhesion to plant leaves and fungal cell wall integrity/oxidative response, respectively. Further RNA-seq analysis on the wild type and *ΔBcgbl1* mutant incubated on the hydrophobic surface allowed identification of the potential downstream target genes, including three novel genes (*BcHsbA1*-*3*) for plant adhesion and virulence. Deletion of each individual *BcHsbA* gene resulted in decreased adhesion and invasion of *B*. *cinerea* on host leaf tissues, suggesting that three *BcHsbA* genes play an important role in fungal virulence. Taken together, this study advanced our understanding of the molecular mechanisms underlying the function of the Gβ-like protein in modulating development and pathogenicity.

## Introduction

*Botrytis cinerea*, the causal agent of gray mold, is an important phytopathogenic fungus with worldwide distribution. It can attack more than 1,400 plant species, including important vegetables, fruits, ornamental and field crops. The gray mold disease not only occurs in greenhouses and fields, but also during storage and transit, resulting in huge economic losses [1]. Because of its significant economic impact, *B*. *cinerea* has been recognized as a model system to study the molecular mechanisms of the infection process of necrotrophic pathogens [2].

Infection of *B*. *cinerea* is initiated by macroconidia, which adhere to plant surfaces and germinate under proper temperature and humid conditions, followed by germ-tube elongation and penetration into plant tissues either directly or through stomata or wounds [3]. For direct penetration, *B*. *cinerea* forms infection-specific structures such as appressorium-like structures developed at the tip of germ tubes or infection cushions formed by hyphae [4]. Adhesion of macroconidia or hyphae to plant cuticles and the subsequent formation of infection structures is the critical early step for successful infection of *B*. *cinerea*. In recent years, numerous virulence factors have been identified during the infection process of *B*. *cinerea*, such as secreted proteases, plant cuticle and cell wall degrading enzymes, botrydial, oxalic acid, necrosis-inducing proteins, small RNAs, and so on [4,5]. The infection process of *B*. *cinerea* is coordinately regulated by multiple conserved signaling pathways, including heterotrimeric G proteins, cyclic AMP-protein kinase A (cAMP-PKA), and mitogen-activated protein (MAP) kinases pathways [6]. However, details of the regulatory network during the infection process are still unclear.

Gβ-like proteins are known as RACK1 (Receptor for Activated C Kinase 1), and they contain seven WD-40 repeats, which are similar to the Gβ subunit. Many studies have demonstrated that Gβ-like protein plays important roles in various biological processes in fungi [7]. For example, in the model fungus *Saccharomyces cerevisiae*, the Gβ-like/RACK1 homolog Cpc-2/Asc1 regulated multiple biological processes, including general amino acid control, pheromone response, mating, and filamentous growth, pseudohyphal development, adhesive growth and dimorphism, cell wall integrity, and stress responses [8–12]. In the human pathogenic fungus *Cryptococcus neoformans*, the Gβ-like homolog Gib2 served as a Gβ in cAMP signaling, and deletion of *Gib2* causes a severe growth defect [13]. Loss of CpcB (a homolog of Gβ-like) causes defects in hyphal growth, conidiophores morphology, cell wall integrity, mycotoxin production, antifungal drug resistance, and virulence in *Aspergillus fumigatus* [14–16]. In the plant pathogen *Ustilago maydis*, deletion of RAK1 (a homolog of Gβ-like) affected cell growth and attenuated cell fusion, cell wall integrity, and pathogenicity [17]. In *Magnaporthe oryzae*, the Gβ-like protein Mip11 regulates cell wall integrity, stress responses, and virulence [18,19]. Disruption of the Gβ-like protein FvGbb2 in *Fusarium verticillioides* affected fumonisin biosynthesis, vegetative growth, and conidiation [20]. Therefore, the Gβ-like proteins are certainly linked to a variety of biological functions such as signal transduction, transcriptional regulation, fungal morphology and virulence [21,22]. However, the regulatory network mediated by the Gβ-like protein and its downstream targets are still undescribed.

In this study, we revealed that the Gβ-like protein Bcgbl1-mediated regulatory network plays an important role in fungal development and pathogenicity of *B*. *cinerea*. Deletion of *Bcgbl1* impaired mycelial growth, conidiation, appressorium formation, stress responses, plant adhesion, abolished formation of infection structures, and completely lost pathogenicity. Loss of *Bcgbl1* resulted in attenuation of the phosphorylation level of MAPKs (Bmp1 and Bmp3). Deletion of *Bcgbl1* caused a significant down-regulation of several virulence genes. Moreover, three hydrophobic surface binding proteins, BcHsbA1, BcHsbA2, and BcHsbA3, may act downstream of the Bcgbl1*-*mediated signaling pathway in regulating adhesion and virulence. These findings provide new insights into the Bcgbl1-mediated MAPK signaling network in regulating development and pathogenicity of *B*. *cinerea*.

## Results

### Identification of Bcgbl1 as a pathogenicity-associated gene in *B*. *cinerea*

**Fig 1:**
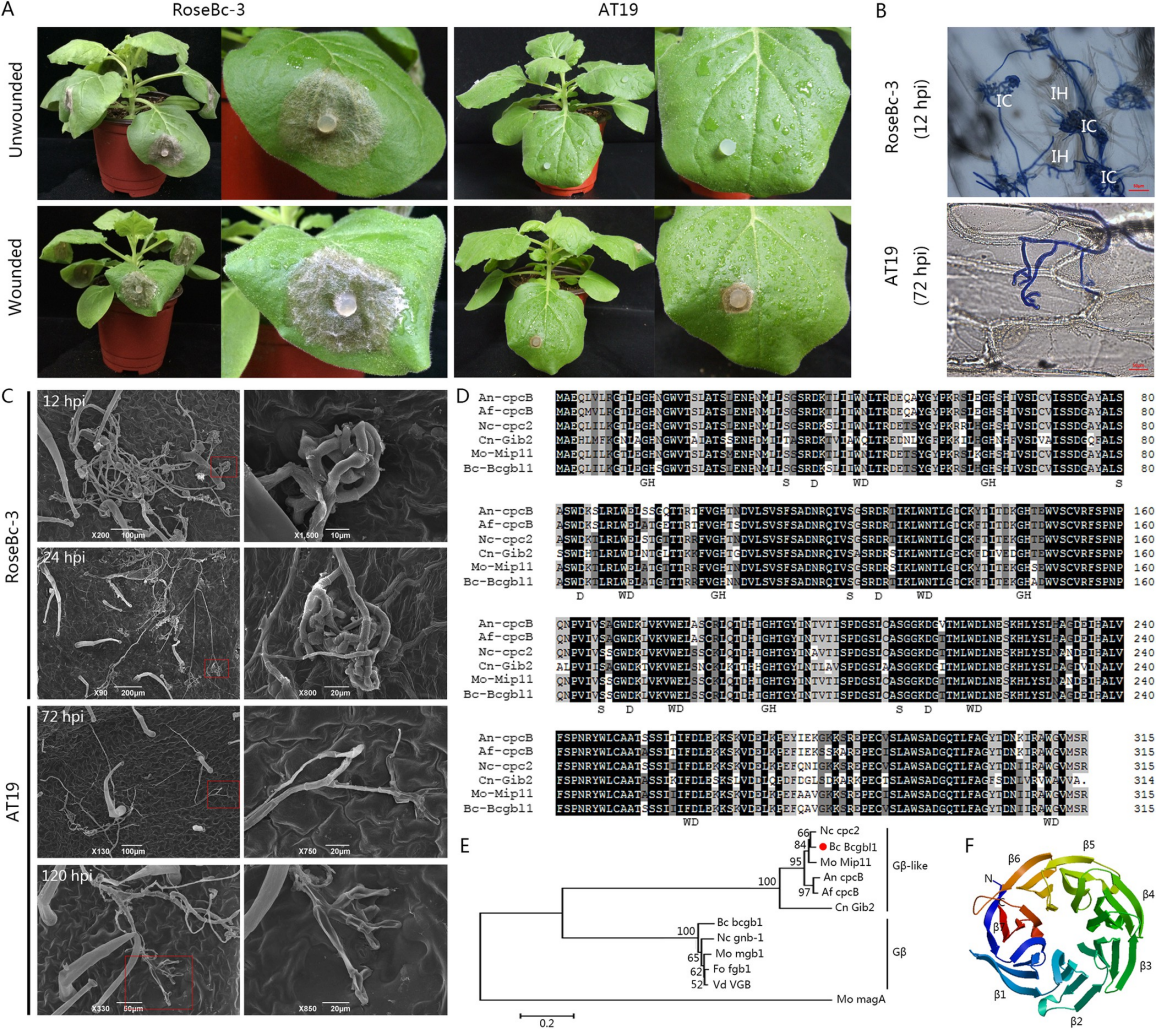
Identification of Bcgbl1 as a pathogenicity factor in *B*. *cinerea*. (A) Pathogenicity test of the WT RoseBc-3 and the T-DNA insertion mutant AT19 on intact tobacco leaves. Mycelial agar plugs (5 mm-diameter) were used as inoculum. Disease symptoms were photographed at 72 hpi. (B) Formation of infection cushion by RoseBc-3 (12 hpi) and AT19 (72 hpi) on the epidermis of onion. Fungal structures were stained with cotton blue. IC: infection cushion, IH: invasive hyphae. (C) Scanning electron micrographs showing the development of infection structures by RoseBc-3 and AT19 on tobacco leaves. (D) Amino acid sequence alignment of Bcgbl1 and orthologues from various fungi (An, *Aspergillus nidulans*; Af, *Aspergillus fumigatus*; Nc, *Neurospora crassa*; Cn, *Cryptococcus neoformans*; Mo, *Magnaporthe oryzae*). All conversed residues are highlighted in black and similar residues are highlighted in gray. The positions of conserved WD and GH repeats, as well as S and D residues in WD proteins, were displayed below the sequences. (E) A neighbor-joining tree of protein Bcgbl1 was constructed with its orthologues in other fungi. The bootstrap values (%) of 1000 repetitions are indicated on branches. (F) The 3D structure of Bcgbl1. Gβ-like protein Gib2 of *Cryptococcus neoformans* was used as the template for the Bcgbl1 model. The seven β-propeller blades were numbered as β1 to β7.

To investigate molecular mechanisms for pathogenicity of *B*. *cinerea*, we previously generated a T-DNA insertion library with *B*. *cinerea* strain RoseBc-3 [23]. A nonpathogenic mutant AT19 was identified, it could not infect the intact (un-wounded) tobacco leaves (Fig 1A). AT19 produced small lesions on wounded leaves, however, the expansion of necrotic lesions was restricted (Fig 1A). Microscopic observation showed that AT19 failed to form infection cushions (ICs) on the epidermis of an onion bulb even at 72 h post inoculation (hpi), while the wild type strain RoseBc-3 formed numerous ICs at 12 hpi (Fig 1B).

To further investigate the structure of the ICs, formation of ICs by RoseBc-3 and AT19 on tobacco leaves was examined by scanning electron microscopy (SEM). RoseBc-3 formed typical ICs at 12 and 24 hpi (Fig 1C). In contrast, AT19 failed to form any ICs at these two time points. At 72 and 120 hpi, AT19 formed a few abnormal ICs with simple branches and shriveled hyphae cells, which appeared lack of penetration capability (Fig 1C).

Southern blot analysis showed that only one copy of T-DNA was inserted into the genome of AT19 (S1A Fig). Then, a 2.0-kb T-DNA flanking sequence in AT19 was obtained using the high-efficient thermal asymmetric interlaced PCR (hiTAIL-PCR) technique [24]. Sequence analysis revealed that the T-DNA was inserted in the 5’-UTR region at the position of 136 bp upstream of the open reading frame (ORF) of the *BC1G_10054* gene (S1B and S1C Fig). This gene was annotated as *Bcgbl1*, which encodes the Gβ-like protein [25]. Furthermore, RT-qPCR analysis showed that the expression of *Bcgbl1* in AT19 was significantly decreased (S1D Fig). These results suggested that the insertion event in the promoter region suppressed expression of *Bcgbl1* in AT19, thereby weakening its pathogenicity.

*Bcgbl1* is 1,242 bp in length, including a 951-bp ORF and two introns, and it was predicted to encode 316 amino acids. Sequence alignment showed that Bcgbl1 shares only 17.39% identity to Gβ protein Bcgb1 [26]. The deduced Bcgbl1 contains a highly conserved seven WD-40 repeat motif (Fig 1D). Phylogenetic analysis showed that Bcgbl1 belongs to the Gβ-like protein (Fig 1E). The predicted three-dimensional structure of Bcgbl1 contains seven β-sheets that are similar to the crystal structure of *C*. *neoformans* Gβ-like protein Gib2 (PDB ID 4D6V) (Fig 1F) [27].

### *Bcgbl1* is required for mycelial growth, sclerotial formation, conidiation and conidial morphology

To determine the function of *Bcgbl1*, the deletion mutants and complemented mutants of *Bcgbl1* were obtained using the wild-type (WT) strain B05.10 as the parent strain (S2A and S2B Fig). Deletion of *Bcgbl1* in Δ*Bcgbl1*-9, Δ*Bcgbl1*-28, and Δ*Bcgbl1*-30 were confirmed by PCR and Southern blotting (S2C and S2D Fig), and complementation of *Bcgbl1* in Δ*Bcgbl1*-30C was verified by RT-qPCR (S2E Fig).

**Fig 2:**
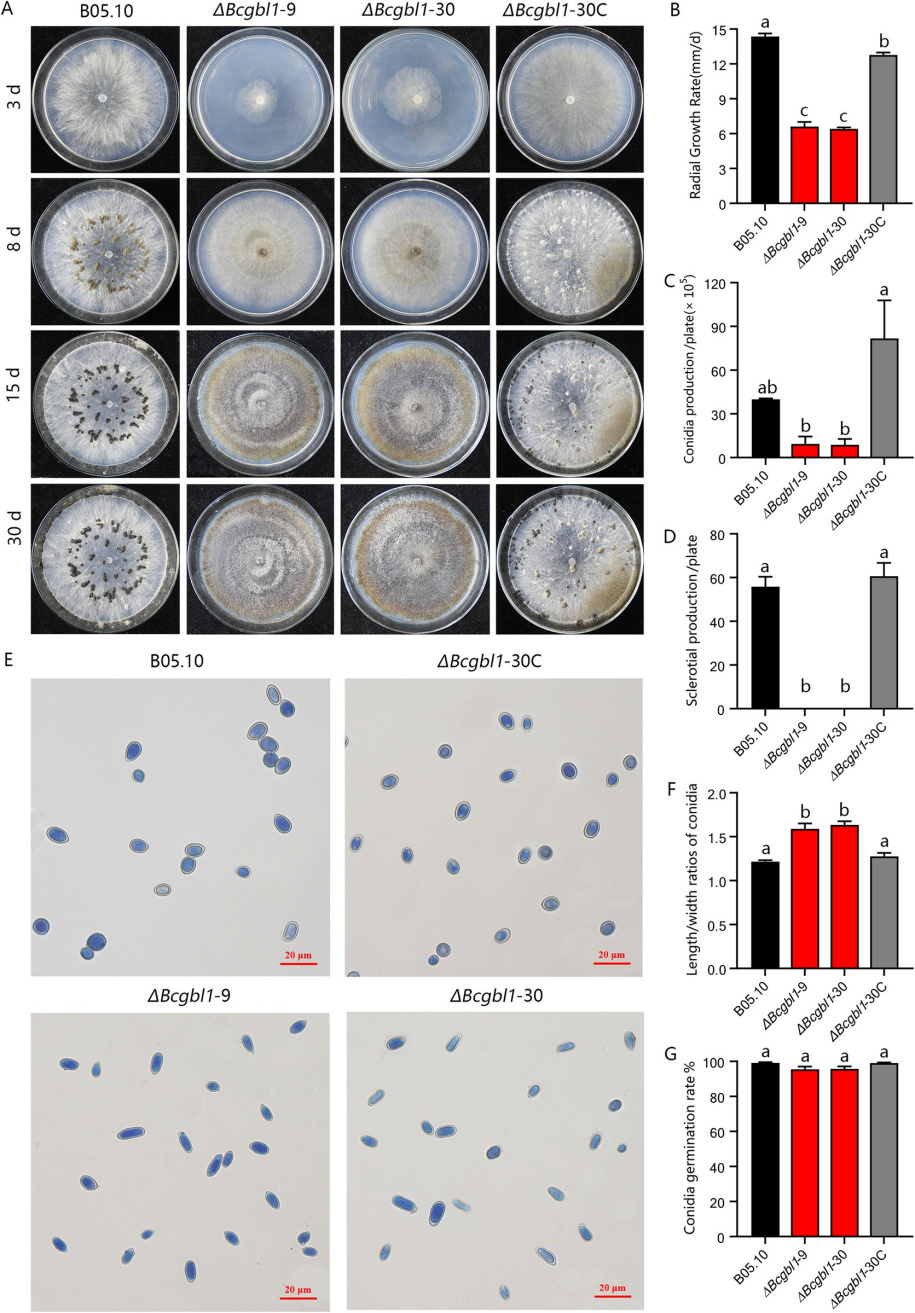
Bcgbl1 is required for mycelial growth, sclerotial formation, conidiation, and conidial morphogenesis. (A) Colony morphology of different strains on PDA medium at 20°C in the dark for 3 to 30 days. (B, C, D) Mycelial growth rates (B), macroconidial production (15 d) (C) and sclerotial production (15 d) (D) of different strains. (E) Morphology of macroconidia produced by different strains (15 d). (F, G) The length-to-width ratios of macroconidia (n = 50) (F) and conidial germination rates (PDA, 20°C, 9 hpi) (G) of different strains. Means ± standard errors labeled with the same letter in each histogram are not significantly different (Least Significant Difference Test, *P* > 0.05).

Mycelial growth and sclerotial formation by the WT and the mutants were examined. While the WT and complemented strains showed normal mycelial growth, conidiation and formed black sclerotia on PDA after incubation for 2 weeks (20°C), the Δ*Bcgbl1* mutants showed reduced mycelial growth and conidiation, and failed to produce sclerotia under the same incubation condition (Fig 2A–2D). Morphology of macroconidia also changed in the Δ*Bcgbl1* mutants. The macroconidia formed by the Δ*Bcgbl1* mutants appeared more elliptical than those formed by the WT and the complemented strains (Fig 2E). The length-to-width ratio of the macroconidia was 1.58 for the Δ*Bcgbl1* mutants, and the value was higher than the WT (1.25) and the complemented strain (1.21) (Fig 2F). Morphological change did not affect macroconidial germination since there was no significant difference in germination rates among the Δ*Bcgbl1* mutants, the WT and complemented strains (Fig 2G). These results showed that Bcgbl1 plays an important role in mycelial growth, sclerotial formation, conidiation, and macroconidial morphology, but is dispensable for macroconidial germination.

### *Bcgbl1* is essential for infection and pathogenicity

**Fig 3:**
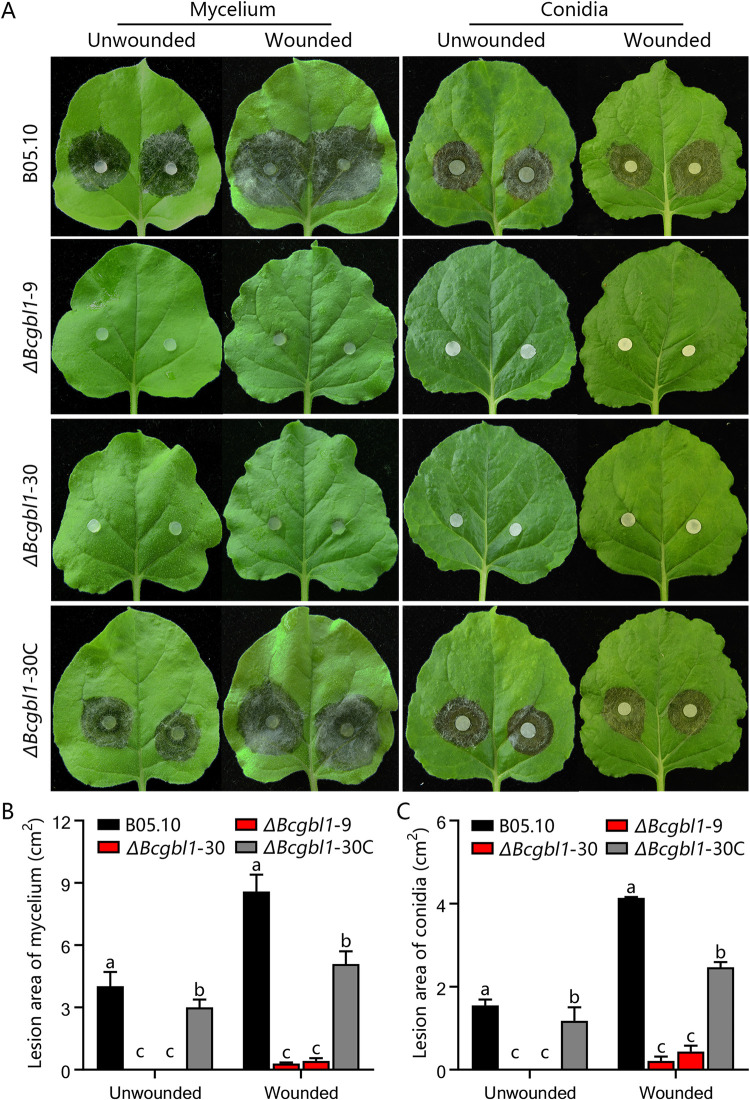
Bcgbl1 is essential for infection and pathogenicity. (A) Pathogenicity test of different strains on tobacco leaves. Mycelial agar plugs (5 mm-diameter) or macroconidial suspension (1 × 10^5^ conidia/mL, 20 μL in half strength of PDB) of each strain were inoculated on unwounded and wounded tobacco leaves, maintained at 20°C under high humid condition for 72 h. (B) Histogram showing average lesion size caused by different strains. Means ± standard errors labeled with the same letter are not significantly different (Least Significant Difference Test, *P* > 0.05).

To investigate the role of *Bcgbl1* in pathogenicity of *B*. *cinerea*, infection bioassays were performed on tobacco leaves *in vitro* with mycelial plugs and macroconidia as inoculum. When mycelium was used as inoculum, the Δ*Bcgbl1* mutants were nonpathogenic on unwounded leaves, while the WT and complemented strains caused severe infection (Fig 3A). The Δ*Bcgbl1* mutants could infect the wounded leaves, but the lesion size was significantly smaller than that formed by the WT and complemented strains (Fig 3A and 3B). When macroconidia were used as inoculum, macroconidia of the Δ*Bcgbl1* mutants failed to cause any symptoms on the intact leaves, and produced small lesions on the wounded leaves with the lesion size significantly smaller than that caused by the WT and complemented strains (Fig 3A and 3B). This demonstrated that *Bcgbl1* is essential for pathogenicity, particularly for penetration and also for lesion expansion in *B*. *cinerea*.

### *Bcgbl1* is required for formation of infection structures

**Fig 4:**
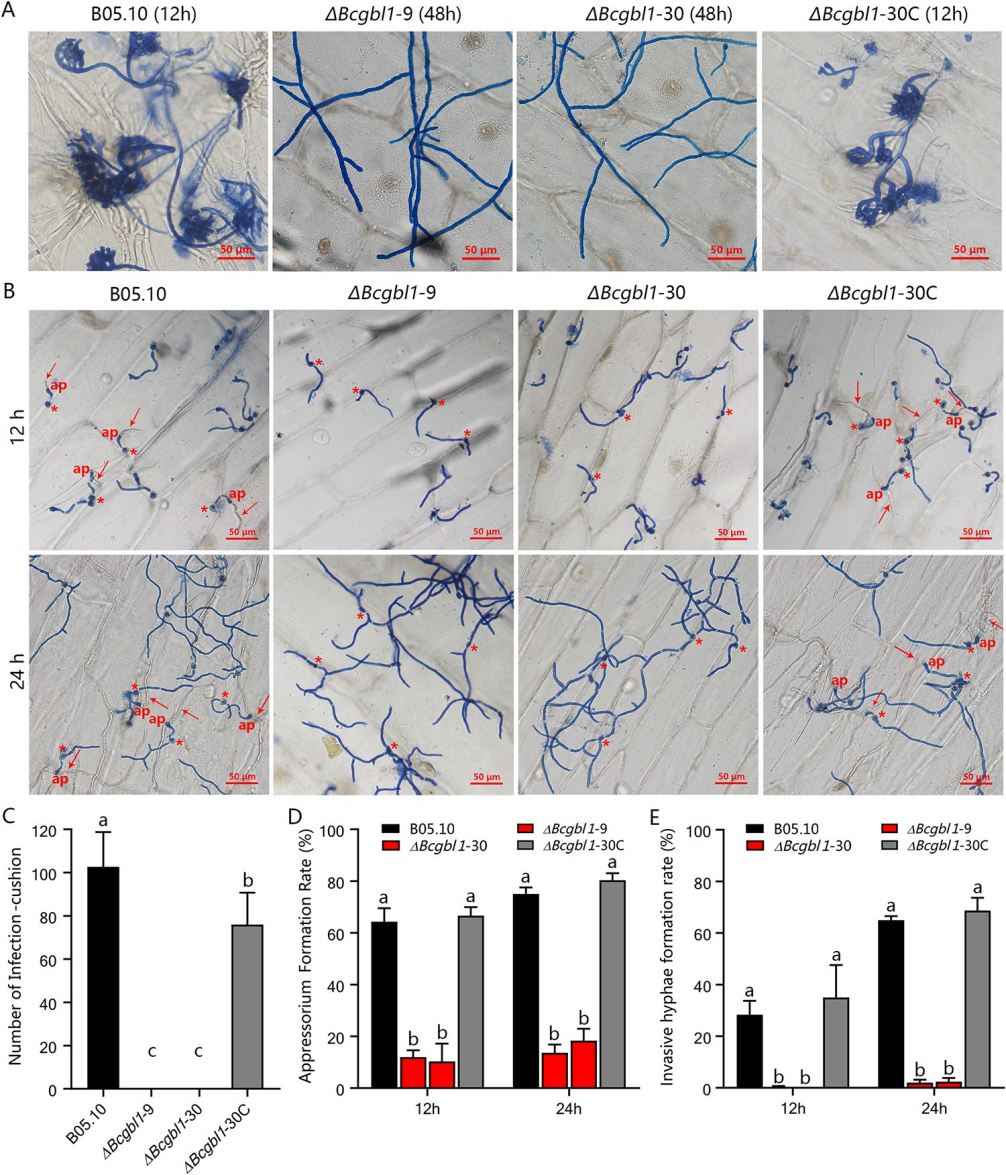
Bcgbl1 is required for formation of infection structures. (A) Formation of infection cushions around the mycelial agar plugs of different strains on onion epidermis. (B) Formation of appressorium-like structures at the tips of germ-tubes from the macroconidia of different strains on onion epidermis. *, macroconidium. ap, appressorium-like structure. Arrows indicate invasive hyphae. (C, D, E) Three histograms showing production of infection cushions, appressorium-like structures and invasive hyphae by different strains on onion epidermis, respectively. Means ± standard errors labeled with the same letter are not significantly different (Least Significant Difference Test, *P* > 0.05).

To validate whether *Bcgbl1* is required for the formation of infection structure, penetration assays were performed on onion epidermal cells with mycelial plugs and macroconidia as inoculum. In the mycelium-inoculation test, abundant infection cushions (ICs) were formed by the WT and the complemented strains at 12 hpi. In contrast, no ICs were formed by the Δ*Bcgbl1* mutants even at 48 hpi (Fig 4A and 4C). In macroconidia-inoculation, appressorium-like structures and invasive hyphae were produced by the WT and complemented strains at 12 and 24 hpi, but were not produced by the Δ*Bcgbl1* mutants even at 36 and 48 hpi (Fig 4B, 4D and 4E). These results showed that *Bcgbl1* is required for formation of infection structures.

### Exogenous cAMP could not restore the defects of the Δ*Bcgbl1* mutants

**Fig 5:**
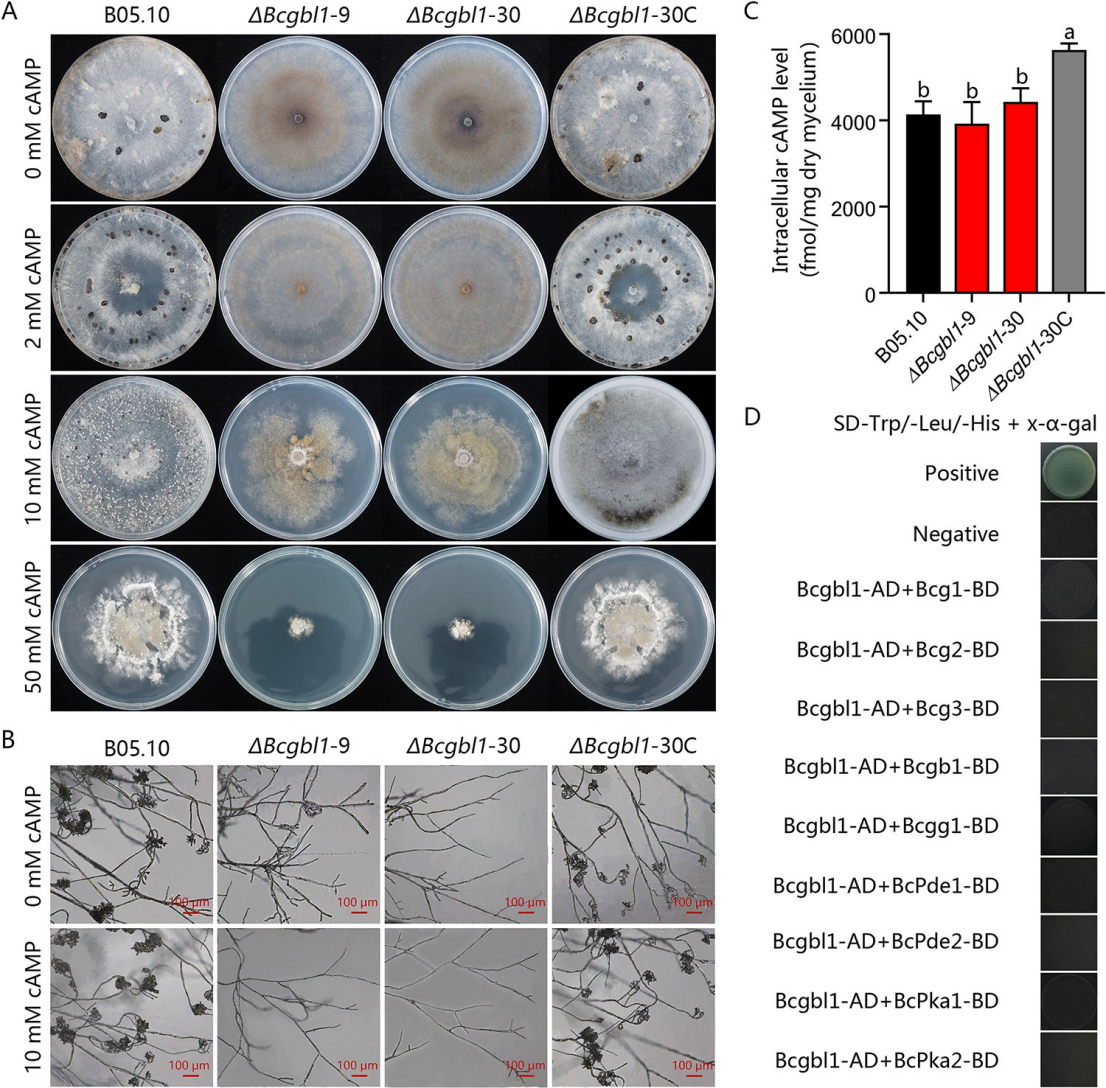
Exogenous cAMP could not restore the defects of the Δ*Bcgbl1* mutants. Exogenous supply of cAMP fails to rescue the defects in morphology (A) and formation of infection cushions (B) of the Δ*Bcgbl1* mutants. (C) Measurement of intracellular cAMP levels in the mycelia of different strains cultured in PDB for 2 days. Two biological repetitions each with three replicates were assayed. Means ± standard errors labeled with the same letter are not significantly different (Least Significant Difference Test, *P* > 0.05). (D) Yeast two-hybrid analysis of Bcgbl1 and the components in the cAMP-signaling pathway. The pairs of plasmids pGBKT7-53/pGADT7-T and pGBKT7-Lam/pGADT7-T served as the positive and negative controls, respectively.

A previous study showed that disruption of the Gβ-like protein gene *MoMip11* in *M*. *oryzae* significantly reduced the intracellular cAMP level, and exogenous supply of cAMP can partially suppress the defect [19]. To test whether Bcgbl1 is involved in the cAMP signaling pathway, we amended PDA with cAMP at 2, 10, and 50 mM, and examined sclerotial formation and conidiation by the Δ*Bcgbl1* mutants. The exogenously applied cAMP did not rescue the defects of the Δ*Bcgbl1* mutants (Fig 5A). We tested the effect of exogenously adding cAMP (10 mM) to the mycelia on formation of ICs. Results showed that the exogenous addition of cAMP failed to rescue formation of ICs by the Δ*Bcgbl1* mutants (Fig 5B). In addition, exogenous addition of IBMX, a phosphodiesterase inhibitor that increases intracellular levels of cAMP, also failed to rescue the reduced mycelial growth of the Δ*Bcgbl1* mutants (S3 Fig). Furthermore, we measured the intracellular cAMP level in mycelia of the WT, Δ*Bcgbl1* mutants and complemented strains. The result showed that there was no significant difference in the content of the intracellular cAMP between the WT and the Δ*Bcgbl1* deletion mutants (Fig 5C).

Since the Gβ-like protein MoMip11 of *M*. *oryzae* interacts with Gα protein MoMagA [19], we further examined the possible interaction of Bcgbl1 with cAMP signaling pathway proteins using yeast two-hybrid assays. The results showed that Bcgbl1 did not interact with Gα protein (Bcg1, Bcg2, and Bcg3), Gβ (Bcgb1), and Gγ (Bcgg1), or any components (BcPde1, BcPde2, BcPKA1, BcPKA2) in the cAMP signaling pathway (Fig 5D).

### Deletion of *Bcgbl1* reduces phosphorylation levels of Bmp1 and Bmp3

**Fig 6:**
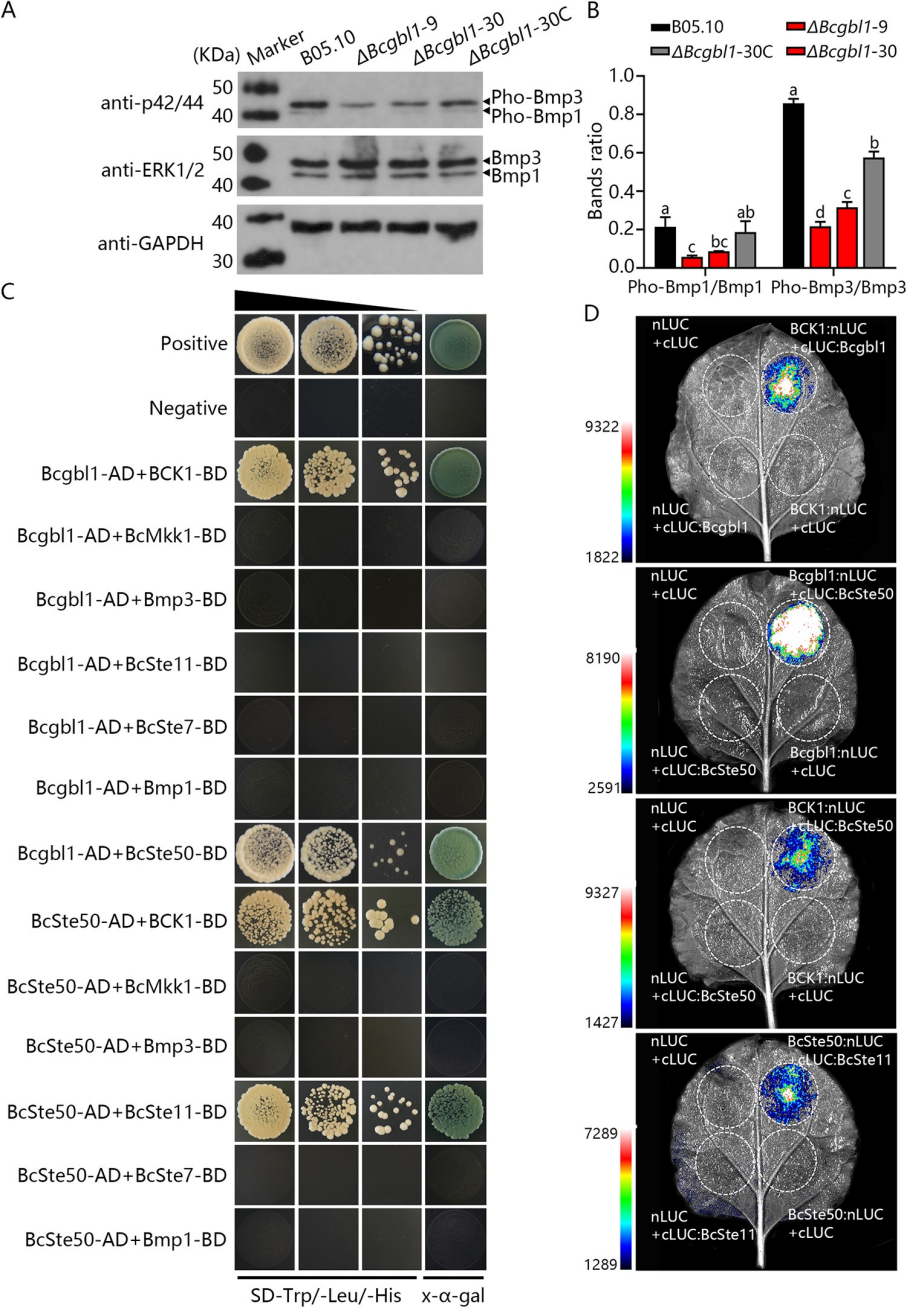
Bcgbl1 regulates Bmp1 and Bmp3 phosphorylation in the mycelia of *B*. *cinerea*. (A) Comparison of the phosphorylation levels of MAPK Bmp1 and Bmp3 in different strains. Bmp1, Bmp3 and the phosphorylated proteins were detected using the ERK1/2 and phospho-p44/42 MAPK antibodies, respectively. (B) Bands ratio of the phosphorylated Bmp1 and Bmp3 bands for each strain is relative to that of the Bmp1 and Bmp3 bands, respectively. Image J software version 1.54d was used to quantify the protein bands. Means ± standard errors labeled with the same letter are not significantly different (Least Significant Difference Test, *P* > 0.05). (C) Yeast two-hybrid assay of Bcgbl1 and the BcSte11-BcSte7-Bmp1/BCK1-BcMkk1-Bmp3 cassette. The pairs of plasmids pGBKT7-53/pGADT7-T and pGBKT7-Lam/pGADT7-T served as the positive and negative controls, respectively. (D) Luciferase complementation assays to confirm the interaction between Bcgbl1 and BcSte50 or BCK1, BcSte50 and BCK1 or Ste11. The proteins were expressed in *N*. *benthamiana* leaves by *Agrobacterium tumefaciens* infiltration, and fluorescence was observed after 48 h.

In *B*. *cinerea*, the mitogen-activated protein (MAP) kinases (Bmp1 and Bmp3) are required for surface recognition and host penetration [28–31]. To investigate the relationship between MAP kinases (Bmp1 and Bmp3) and Bcgbl1, phosphorylation profiling of Bmp1 and Bmp3 was assayed with the anti-TpEY specific antibody. Compared with the WT and complemented strains, the Δ*Bcgbl1* mutants had significantly reduced phosphorylation levels of Bmp1 and Bmp3 (Fig 6A and 6B), suggesting that Bcgbl1 plays an important role in regulating Bmp1 and Bmp3 phosphorylation.

Subsequently, we tested whether Bcgbl1 directly interacts with any Bmp1 and Bmp3 signaling component proteins through yeast two-hybrid assays. Results showed that Bcgbl1 did not directly interact with Bmp1 and the upstream proteins BcSte11 and BcSte7. In the Bmp3 MAP kinase cascade, Bcgbl1 directly interacted with the putative MAPKKK BCK1, but not with BcMkk1 and Bmp3. Furthermore, we found that Bcgbl1 directly interacted with BcSte50 (Fig 6C). BcSte50 protein is speculated to be an adapter protein of the BcSte11-BcSte7-Bmp1 cascade regulating various aspects of growth, stress response, and plant infection [31]. We further tested the interactions between BcSte50 and MAPK (Bmp1 and Bmp3) signaling component proteins using yeast two-hybrid assays. As expected, BcSte50 directly interacted with BcSte11 and BCK1 (Fig 6C). Finally, we performed luciferase complementation assays (LCA) to further confirm the interaction between these proteins in tobacco leaves. LCA results strongly indicate that Bcgbl1 directly interacts with BcSte50 and BCK1, and BcSte50 directly interacts with BcSte11 and BCK1 (Fig 6D). These results suggest that Bcgbl1 regulates Bmp1 and Bmp3 phosphorylation likely through BcSte50.

### *Bcgbl1* affects tolerance against high salt and oxidative stress

**Fig 7:**
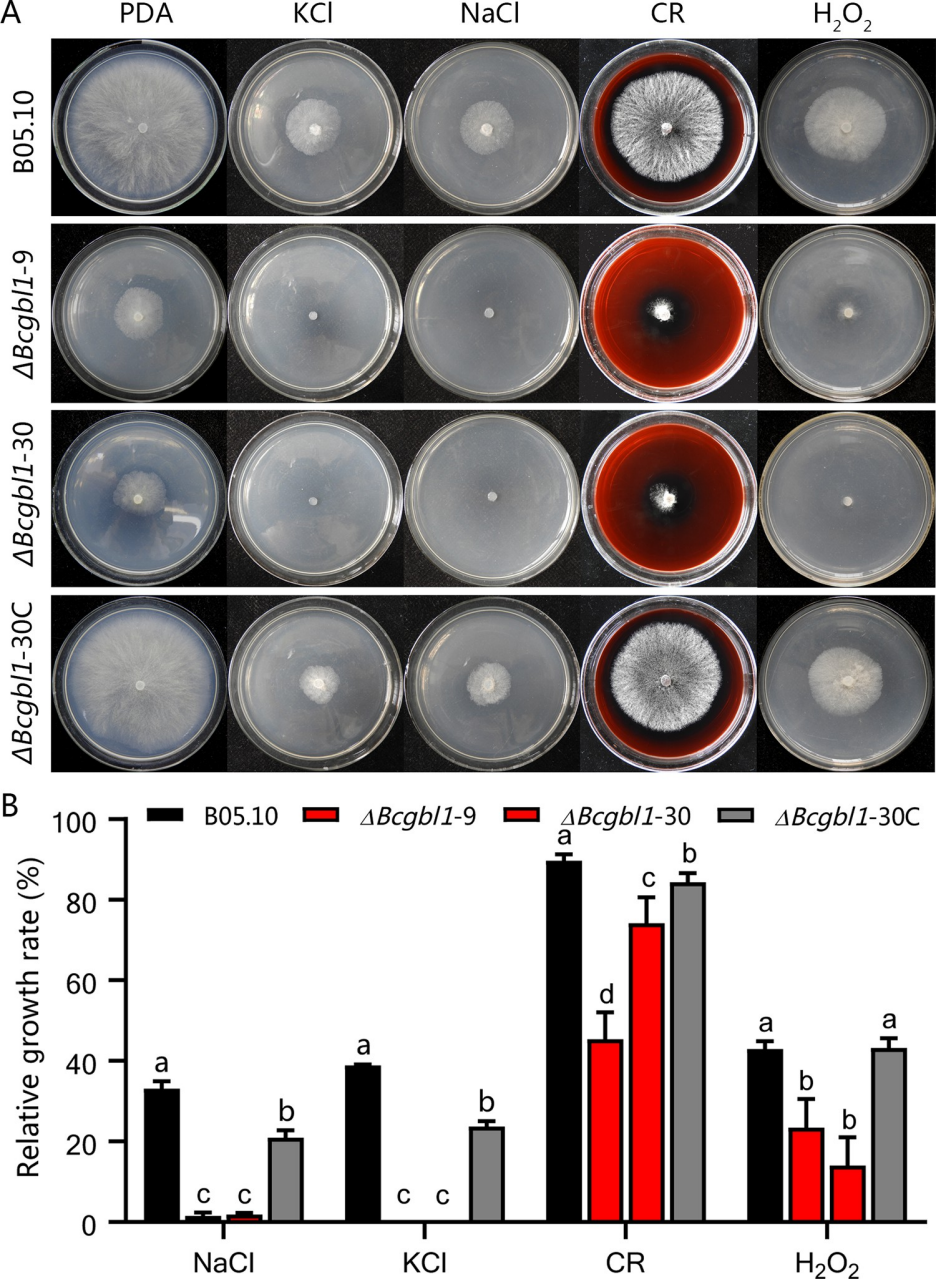
Bcgbl1 affects tolerance against high salt and oxidative stresses. (A) Sensitivity test of the indicated strains to salt stress agents NaCl (1 mol/L) and KCl (1 mol/L), cell wall disturbing agent Congo Red (CR, 0.3 mg/mL), and oxidative stress agent H_2_O_2_ (5 mmol/L). (B) The relative mycelial growth rates of different strains in the presence of various stress chemicals against the controls on PDA alone. Means ± standard errors labeled with the same letter are not significantly different (Least Significant Difference Test, *P* > 0.05).

Previous studies showed that the BCK1-BcMkk1-Bmp3 cascade is involved in regulating the cell wall integrity signaling pathway [30]. Considering that Bmp3 phosphorylation was reduced in the Δ*Bcgbl1* mutants, we tested whether Bcgbl1 also regulates the cell wall stress tolerance in *B*. *cinerea*. The Δ*Bcgbl1* mutants were almost unable to grow on PDA amended with NaCl or KCl (both at 1 mol/L), and were more sensitive to the cell wall disturbing agent Congo Red (0.3 mg/mL) and the oxidative agent H_2_O_2_ (5 mmol/L) than the WT and complemented strains (Fig 7A and 7B). These results showed that Bcgbl1 participates in regulating the cell wall stress response in *B*. *cinerea*. Collectively, our findings suggest that Bcgbl1 is involved in regulating the phosphorylation of Bmp3 in response to cell wall and oxidative stress in *B*. *cinerea*.

### Bcgbl1 globally regulates expression of hydrophobic surface-induced genes

**Fig 8:**
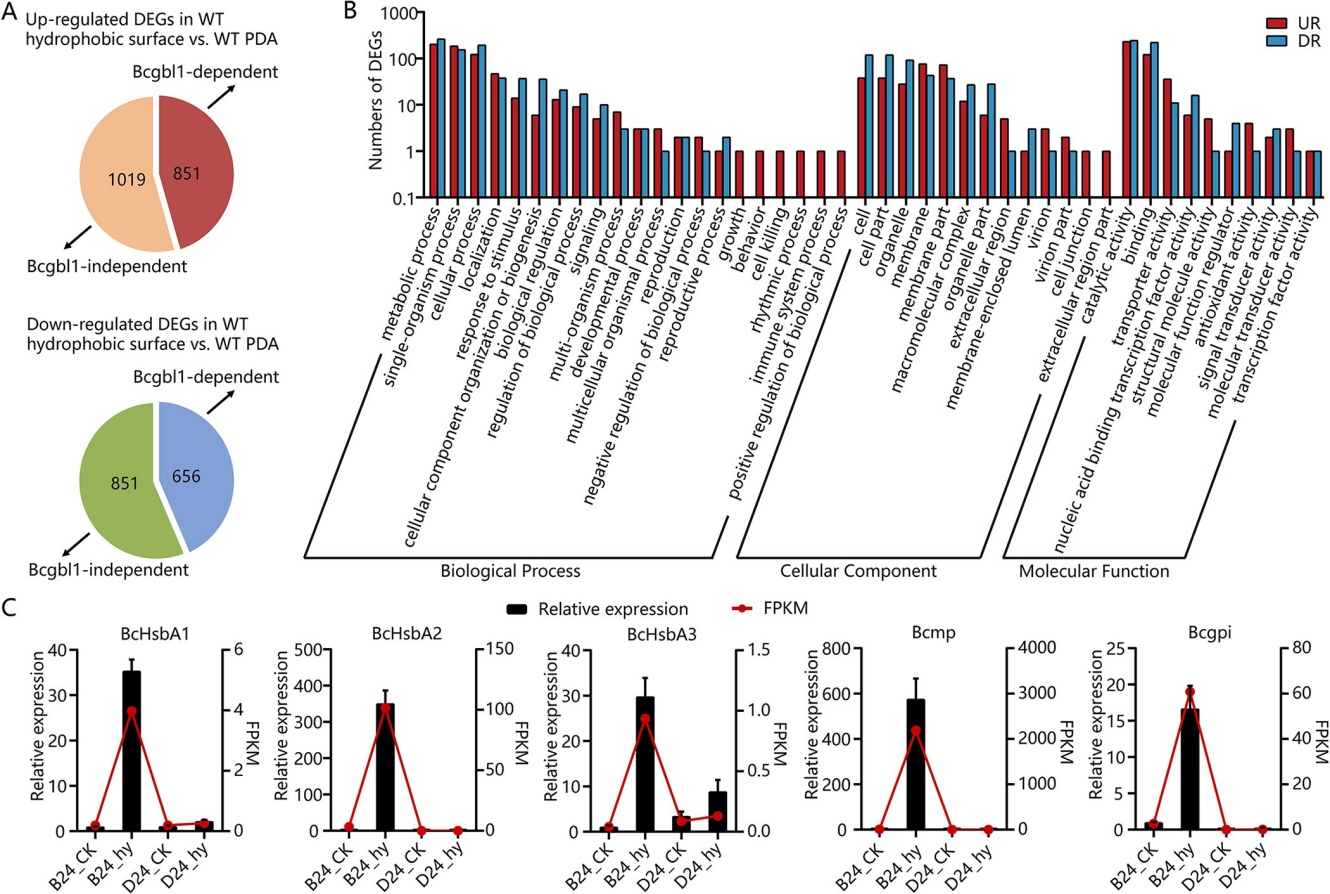
Bcgbl1 globally regulates expression of hydrophobic surface-induced genes. (A) Schematic diagram showing number of differentially expressed genes (DEGs) induced by hydrophobic surface in Bcgbl1-dependent manner. On the basis of the comparison of fold change values between WT and Δ*Bcgbl1*-30 (fold change values of gene expression level in hyphae on the hydrophobic surface relative to hyphae in PDA cultures), Bcgbl1-dependent genes were identified when the rate of fold change increase or decrease in the Δ*Bcgbl1*-30 mutant was less than 50% that in WT, the remaining genes were identified as Bcgbl1-independent. (B) Gene ontology (GO) classification of Bcgbl1-dependent up-regulated and down-regulated genes. (C) RT-qPCR validation of the RNA-seq data showed that up-regulation expression of *BcHsbA1*, *BcHsbA2*, *BcHsbA3*, *Bcmp*, and *Bcgpi* on the hydrophobic surface was Bcgbl1-dependent. These five Bcgbl1-regulated genes all contain the putative Ste12 binding sites (5’-TGAAACA-3’) (S5 Fig) on their promoter region. B for the WT B05.10, D for the deletion mutant Δ*Bcgbl1*-30, CK means inoculation on PDA to stimulate vegetative growth, hy means inoculation on hydrophobic surface to simulate the condition for infection cushion formation. B24_CK and D24_CK, represent the WT and Δ*Bcgbl1*-30 grew on PDA for 24 h (20°C), respectively; B24_hy and D24_hy, represent the WT and Δ*Bcgbl1*-30 grew on the hydrophobic surface for 24 h (20°C), respectively.

Since we had observed Δ*Bcgbl1* mutant defect in infection cushions formation during host epidermal cells infection, we performed RNA-seq analysis on the WT and Δ*Bcgbl1* mutant incubated on the hydrophobic surface (plastic slide to mimic plant surface induction) for 24 h. Mycelium of the WT and Δ*Bcgbl1* mutant incubated on PDA for 24 h were used as controls. Upon hydrophobic surface induction, 1870 and 1507 genes showed at least 2.0-fold up-regulation and down-regulation in the WT compared to the expression level in the WT grown on PDA, respectively (Fig 8A). Comparison of up- and down-regulation folds of the differentially expressed genes (DEGs) in Δ*Bcgbl1* and WT showed that in Δ*Bcgbl1* mutant, 851 and 656 genes were up- or down-regulated (less than 50% of the WT level), these genes were considered as Bcgbl1-dependent (Fig 8A). Statistical analysis showed that 44.63% of the hydrophobic surface-induced genes are regulated by Bcgbl1. Gene ontology (GO) analysis showed that the Bcgbl1-dependent up-regulated genes were mainly enriched in catalytic activity, metabolic process, single-organism process, cellular process, and binding (Fig 8B). They may be involved in adhesion, formation of infection cushions, and early infection. GO analysis also showed that the Bcgbl1-dependent down-regulated genes were mainly enriched in metabolic process, catalytic activity, binding, cellular process, and single-organism process (Fig 8B). Taken together, the RNA-seq analysis showed that nearly half of the hydrophobic surface-induced genes were regulated by Bcgbl1, thus highlighting the global role of *Bcgbl1* in regulation of gene expression upon hydrophobic surface induction.

Since Bmp1 phosphorylation was reduced in the Δ*Bcgbl1* mutants, and the defect phenotype of the Δ*Bcgbl1* mutants was similar to that of the Δ*bmp1* mutant [31], suggesting that Bcgbl1 may regulate the Bmp1 signaling pathway. As a downstream protein of Bmp1, the transcription factor BcSte12 has been reported to regulate development and pathogenicity in *B*. *cinerea* [31]. We speculated that Bcgbl1 also affects BcSte12 and its downstream target genes in *B*. *cinerea*. As expected, the yeast two-hybrid result showed that Bmp1 directly interacts with BcSte12 (S4A Fig). The previous study showed that *Bcgas2* is a downstream target of Bmp1 and BcSte12, and its expression is controlled by Bmp1 and BcSte12 [31]. Therefore, we examined the expression of *Bcgas2* in the WT and Δ*Bcgbl1* mutant. RT-qPCR result showed that *Bcgas2* is significantly down-regulated in the Δ*Bcgbl1* mutant (S4B Fig), indicating that the expression of *Bcgas2* is also regulated by Bcgbl1.

Among Bcgbl1-regulated genes, we selected five genes for detailed study, based on their promoter region containing the putative Ste12 binding sites (5’-TGAAACA-3’) [32] (S5 Fig). These genes included three genes encoding the hydrophobic surface binding proteins [*BcHsbA1* (Bcin03g08230), *BcHsbA2* (Bcin04g06380), and *BcHsbA3* (Bcin10g04570)], *Bcmp* (Bcin12g06300) coding for metallopeptidase, and *Bcgpi* (Bcin04g00460) coding for the glycosylphosphatidylinositol (GPI)-anchored protein. Consistent with RNA-seq analysis, RT-qPCR showed that the expression of these five genes in the WT was dramatically increased when it was inoculated onto the hydrophobic surface. However, the fold induction of these five genes in response to the hydrophobic surface was significantly reduced in the Δ*Bcgbl1* mutant (Fig 8C). These results suggested that the up-regulation of these five genes in response to the hydrophobic surface requires the presence of functional Bcgbl1.

### Three Bcgbl1-regulated genes, *BcHsbA1*, *BcHsbA2*, and *BcHsbA3*, are involved in plant adhesion and virulence

**Fig 9:**
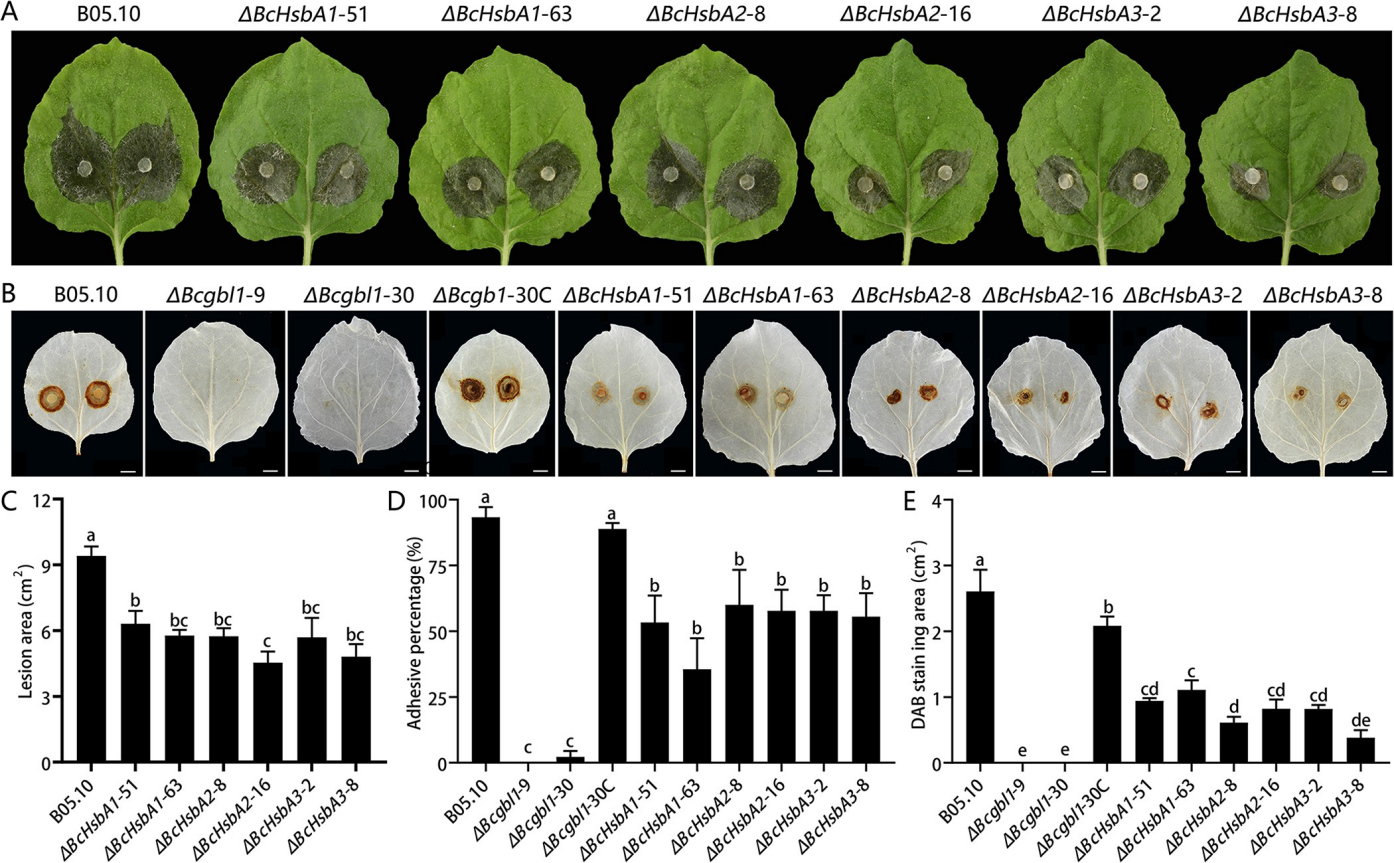
Three Bcgbl1-regulated genes (*BcHsbA1*, *BcHsbA2*, *BcHsbA3*) are involved in plant adhesion and virulence. (A) Virulence of Δ*BcHsbA1*, Δ*BcHsbA2*, and Δ*BcHsbA3* on tobacco leaves (20°C, 72 h). (B) DAB staining of the tobacco leaves inoculated with different strains at 24 hpi, brown color indicated H_2_O_2_ generation in dead cells infected with a given fungal strain. DAB (1 mg/mL) was taken up by the detached leaves at the beginning of inoculation. Scale bar, 1 cm. (C) Lesion size on tobacco leaves caused by different strains. (D) Adhesion percentage of mycelial plugs of different strains at 9 hpi on tobacco leaves (20°C). (E) DAB staining area of the tobacco leaves inoculated with different strains at 24 hpi. Image J software version 1.54d (National Institutes of Health, Bethesda, MD, USA) was used to quantify the area of DAB staining of the tobacco leaves. Means ± standard errors labeled with the same letter are not significantly different (Least Significant Difference Test, *P* > 0.05).

To further explore the functions of these five Bcgbl1-dependent genes, these genes were individually deleted and the deletion mutants (Δ*BcHsbA1*, Δ*BcHsbA2*, Δ*BcHsbA3*, Δ*Bcmp*, and Δ*Bcgpi*) were obtained. Colony morphology, mycelial growth, and sclerotial formation of these deletion mutants on PDA were unaffected, compared to the WT (S6A and S6B Fig). Interestingly, three *BcHsbA* deletion mutants (Δ*BcHsbA1*, Δ*BcHsbA2*, Δ*BcHsbA3*) displayed reduced virulence (Fig 9A and 9C). By contrast, deletion of the other two genes (*Bcmp* and *Bcgpi*) did not affect virulence (S7 Fig). These results suggested that the three *BcHsbA* genes, but not *Bcmp* and *Bcgpi*, play important roles in virulence.

Previous study indicated that the HsbA protein secreted by *Aspergillus oryzae* can adhere to hydrophobic surface [33], we investigated whether *Bcgbl1* or the three *BcHsbA* genes regulate the adhesion of *B*. *cinerea* to host leaves. Tobacco leaves were inoculated with mycelial plugs of the WT, the complemented strain, Δ*Bcgbl1* and Δ*BcHsbA* mutants. The adhesion percentage of the WT and complemented strains was 93% and 87%, respectively. Interestingly, almost all mycelial plugs of the Δ*Bcgbl1* mutants fell off from the tobacco leaves (Fig 9D). These results clearly showed that the Δ*Bcgbl1* mutant mycelium almost lost the ability to adhere to tobacco leaves, which might explain their failure of penetration and complete loss of pathogenicity. Similarly, three *BcHsbA* mutants showed reduced adhesion percentage on tobacco leaves compared to the WT, but the decline was less than that of the Δ*Bcgbl1* mutants (Fig 9D). Consistent with the virulence results, at 24 hpi, we found that the DAB staining in the treatments of the three *BcHsbA* mutants were significantly reduced, and, interestingly, no visible DAB staining was observed in the leaves inoculated with the Δ*Bcgbl1* mutants (Fig 9B and 9E). Furthermore, we tested whether *BcHsbA* genes associate with the response to cell wall stress. The Δ*BcHsbA* mutants exhibited similar sensitivity to cell wall agents as the WT (S8A and S8B Fig). Taken together, these observations suggest that three Bcgbl1-regulated genes (*BcHsbA*1-3) are involved in fungal adhesion and virulence.

## Discussion

**Fig 10:**
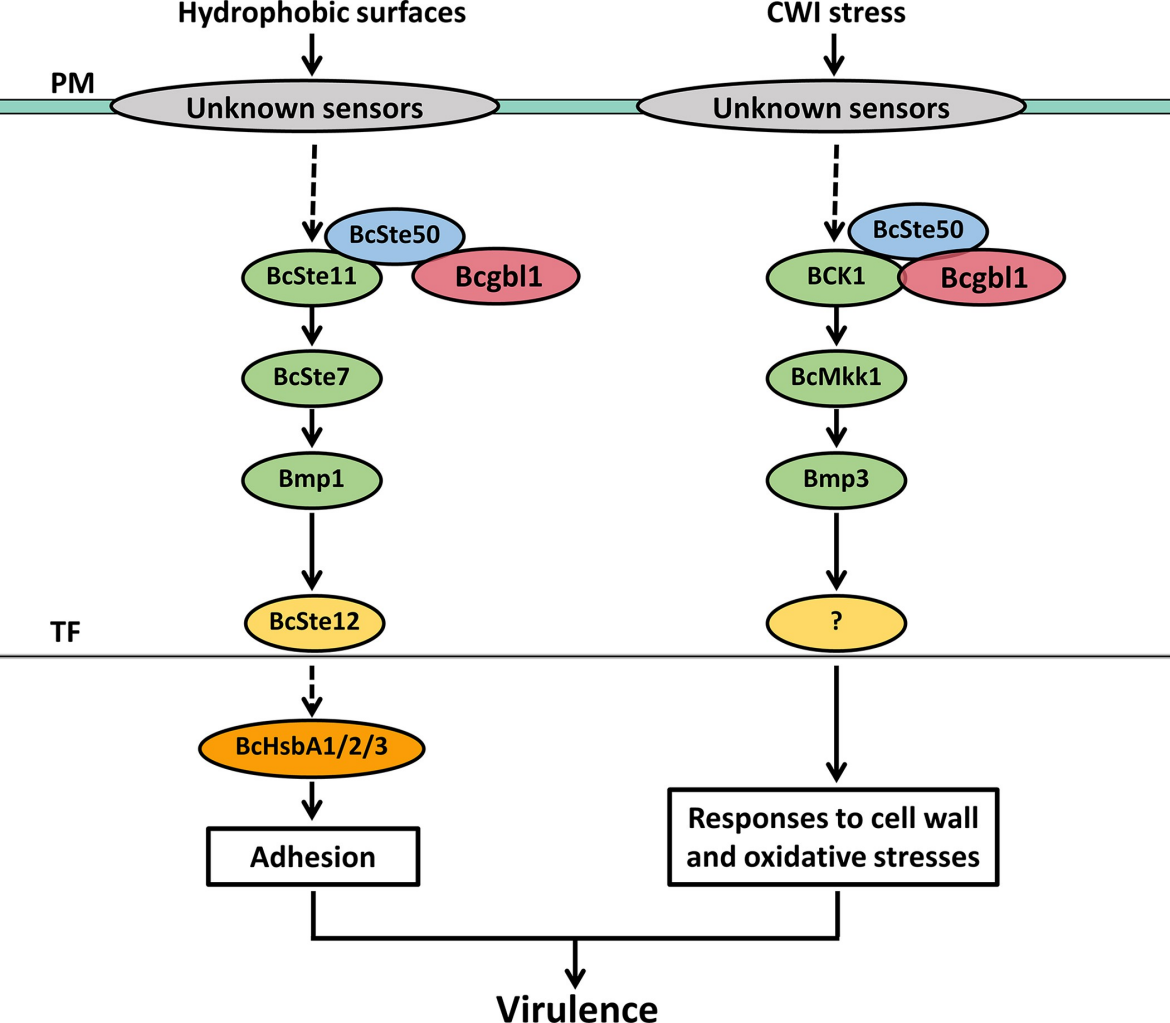
A proposed model for the roles of Bcgbl1 in regulation of virulence of *B*. *cinerea*. Bcgbl1 positively regulates pathogenicity mainly through the MAPK signaling pathways, which control growth, conidiation, sclerotial formation, infection structures formation, plant adhesion, and responses to cell wall and oxidative stresses. On the one hand, Bcgbl1 positively regulates Bmp1 phosphorylation mediated by BcSte50, resulting in activation of the downstream target genes, including *BcHsbA1/2/3* by the Bmp1-BcSte12 regulatory pathway, thereby regulation of plant adhesion and virulence. On the other hand, Bcgbl1 mediates phosphorylation of Bmp3 through BCK1 or BcSte50, which controls responses to cell wall and oxidative stresses. Dotted lines, unidentified; PM, plasma membrane; TF, transcription factor; CWI, cell wall integrity.

The regulatory networks of fungal infection-related morphogenesis in *B*. *cinerea* remain largely unexplored. In this study, we demonstrated that Bcgbl1-mediated MAPK phosphorylation plays an important role in regulating infection-related morphogenesis and pathogenicity of *B*. *cinerea*. Bcgbl1 positively regulates the phosphorylation levels of Bmp1 and Bmp3 likely through BcSte50. The enhanced phosphorylation level of Bmp1 may further induce expression of the downstream target genes *BcHsbA*1-3 to regulate adhesion and virulence (Fig 10).

Bcgbl1 is the homolog of *S*. *cerevisiae* Gβ-like protein Asc1, which regulates multiple biological processes, including general amino acid control, pheromone response, mating, filamentous growth, pseudohyphal development, translation regulation, and fidelity, cell size, cell wall integrity, stress response, and cAMP/PKA and MAPK pathways [7,9–12]. In *M*. *oryzae*, the Gβ-like protein MoMip11 is also a multifunctional protein that participates in regulation of hyphal growth, asexual and sexual development, cell wall integrity, stress responses, cAMP and MAPK signaling, infection, and pathogenicity [18,19]. Similarly, Bcgbl1 in *B*. *cinerea* plays multiple roles in hyphal growth, conidiation, sclerotial production, stress adaptation, formation of infection structures, adhesion, and pathogenicity *via* genome-wide orchestration of expression of the genes associated with fungal pathogenesis with highlights of regulation of the MAPK signaling pathways.

Gβ-like protein contains a seven-bladed β-propeller structure that is similar to the Gβ subunit. In several fungi, the Gβ-like protein directly interacts with one of the Gα subunits functioning as a Gβ subunit involved in regulation of cAMP signaling, such as in *S*. *cerevisiae* [9], *Cryptococcus neoformans* [13], and *M*. *oryzae* [19]. In *M*. *oryzae*, the Gβ-like protein MoMip11 positively regulates the cAMP level by interacting with MoMagA and MoPdeH [19]. MoMip11 has a positive role in the regulation of intracellular cAMP levels that are essential for pathogenicity. MoMip11 also interferes with the interaction between MoRgs7 and activated Gα resulting in a positive role in cAMP signaling. In addition, MoMip11 could partially compensate for lacking of Gβ in appressorium formation. *B*. *cinerea* contains seven genes encoding RGS-like proteins. However, phylogenetic analysis showed that there is no homologous gene of *MoRgs7* in *B*. *cinerea*. This may be the difference in the functions and downstream signaling of Gβ-like proteins between *B*. *cinerea* and *M*. *oryzae*. Intriguingly, in *F*. *verticillioides*, the Gβ-like/RACK1 homolog FvGbb2 does not interact with any G protein subunits [20]. In our study, we failed to establish the interaction between Bcgbl1 and G protein subunits (or components in the cAMP signaling pathway) by yeast two-hybrid assays. Furthermore, deletion of *Bcgbl1* in *B*. *cinerea* did not affect the intracellular cAMP level in mycelia, and exogenous supply of cAMP or IBMX failed to rescue the defects of the Δ*Bcgbl1* mutants. This result suggests that Bcgbl1 is probably not involved in the cAMP signaling pathway in *B*. *cinerea*.

MAP kinase (MAPK) cascades are well known conserved in filamentous fungi playing an important role in regulating various cellular processes, including hyphal growth, stress response, and plant infection [34–36]. Previous studies demonstrated that the Gβ-like/RACK1 protein is involved in regulation of the MAPK signaling pathway [9,18]. In *S*. *cerevisiae*, deletion of the Gβ-like/RACK1 gene *Asc1* leads to a significant increase in the phosphorylation level of both MAPK Kss1 and Slt2 [9,37]. In *M*. *oryzae*, loss of the Gβ-like/RACK1 gene *MoMip11* causes an increase in the phosphorylation level of MAPK Mps1 [18]. However, the phosphorylation level of both MAPKs (Bmp1 and Bmp3) was decreased in the Δ*Bcgbl1* mutants (Fig 6A). Although deletion of the Gβ-like/RACK1 gene in *M*. *oryzae* and *B*. *cinerea* resulted in different levels of MAPK Slt2 (Mps1/Bmp3) phosphorylation, both mutants exhibited defects in cell wall integrity. But the molecular mechanism of how Gβ-like/RACK1 regulates MAPK signaling remains unclear. The Gβ-like/RACK1 proteins have been shown to interact directly with one of the MAPK cascade proteins in fungi, such as in *S*. *cerevisiae* [9] and *M*. *oryzae* [18]. In *S*. *cerevisiae*, the Gβ-like/RACK1 protein Asc1 negatively regulates the Kss1 MAPK pathway by binding to Ste20 [9]. In *M*. *oryzae*, MoMip11 interacts with Mck1 and Ste50 [18]. Furthermore, in *Arabidopsis thaliana*, the Gβ-like/RACK1 protein functions as a scaffold that binds to all three tiers of the MAPK cascade [38]. Similarly, our results also revealed that Bcgbl1 interacts with the putative MAPKKK BCK1 and the MAP kinase adaptor protein BcSte50. Although we did not provide evidence of direct interaction between Bcgbl1 and Bmp1 or the upstream proteins BcSte11 and BcSte7, we found that BcSte50 directly interacted with MAPK upstream proteins, BcSte11, and BCK1, respectively. In *B*. *cinerea*, the Δ*bmp1* mutant showed defects in vegetative growth, macroconidia size, sclerotial formation, and pathogenicity [28,31], which is similar to the Δ*Bcgbl1* mutant. Therefore, our data indicated that Bcgbl1 is an upstream regulator of MAPK cascade, and Bcgbl1 positively regulates the phosphorylation of Bmp1 and Bmp3 likely through BcSte50.

The conserved transcription factor Ste12 acting as one of the key downstream targets of the Fus3/Kss1 MAPK pathway plays an important role in regulating development and pathogenicity [32,39]. Although Ste12 is required for infection in many phytopathogenic fungi, the downstream target genes of Ste12 are still unclear. In the current study, we found that Bmp1 directly interacts with BcSte12. This result suggests that Bcgbl1 may affect BcSte12 and its target genes *via* Bmp1.

We speculate that the downstream target genes of BcSte12 are also regulated by Bcgbl1. For example, *Bcgas2*, a downstream target gene of BcSte12 [31], was significantly downregulated in the Δ*Bcgbl1* mutant. In addition, the deletion mutant of BcSte12 also showed similar phenotypes to the Δ*Bcgbl1* mutants, such as delayed infection, lack of sclerotial formation, and attenuated pathogenicity [31]. Therefore, we compared the transcriptomes between the WT and the Δ*Bcgbl1* mutant, and identified five Bcgbl1-regulated genes harboring the BcSte12-binding site in their promoter regions. However, we did not obtain evidence of the direct interaction between BcSte12 and these five putative downstream target genes by electrophoretic mobility shift assay (EMSA) or yeast one-hybrid (Y1H). In the future, chromatin immunoprecipitation sequencing (ChIP-seq) will be used to directly analyze the regulatory relationship between BcSte12 and the putative downstream genes identified in this study.

Knockout analysis was conducted for these five putative downstream target genes of BcSte12. We showed that three of these genes, the hydrophobic surface binding protein genes (*BcHsbA1-3*), are required for adhesion and virulence. HsbAs are secreted small proteins that are adsorbed to hydrophobic solid materials and involved in their degradation [33,40]. In *M*. *oryzae*, four homologs of *HsbA* were up-regulated during appressorium development, indicating that they may function in appressorium formation and infection [41]. Furthermore, the deletion mutant of the HsbA gene *MoSVP* displayed defects in invasive hyphae formation and virulence, suggesting that *MoSVP* is required for infection at the initial stage [42]. Adhesion of fungal spores and hyphae to plant cuticles is necessary for penetration, which is the determinative factor of successful infection. Several fungal surface proteins have been proven to be involved in the process of fungi adhesion to plant cuticles, such as cutinase and esterase [43]. In this study, we found that *BcHsbAs* were up-regulated when inoculated on a hydrophobic plastic surface after 24 hpi. The fold induction of *BcHsbAs* was largely compromised in the Δ*Bcgbl1* mutant. Bcgbl1 mutant showed a complete loss of the ability of adhesion and pathogenicity partially due to the down-regulated of *BcHsbAs*. Together, these results suggest that *BcHsbAs* are the downstream targets of the Bcgbl1 regulatory pathway, which play an important role in adhesion and pathogenicity in *B*. *cinerea*.

In *B*. *cinerea*, BcSAK1 (the Hog-type MAP kinase) affects vegetative differentiation and tolerance against oxidative and osmotic stresses [44]. Deletion of MST50 (an ortholog of BcSTE50) also affected OSM1 (a HOG1 ortholog in *M*. *oryzae*) phosphorylation [18]. The defects of the Δ*Bcgbl1* mutants in stress responses are likely related to the HOG pathway. Regulation of Bcgbl1 on the HOG pathway needs further investigation.

In conclusion, we demonstrated that the Gβ-like protein Bcgbl1 plays important roles in mycelial growth, sclerotial formation, conidiation, adhesion, formation of infection structures, pathogenicity and stress tolerance through the regulation of two MAPK signaling pathways in *B*. *cinerea*. The identified Bcgbl1-mediated regulatory network controls the fungus to orchestrate its gene expression for fungal adhesion and infection. These findings contribute to a better understanding of the role of Bcgbl1 in fungal development and pathogenicity and provide a novel insight into the Gβ-like protein-mediated regulatory network in fungal infection.

## Materials and methods

### Fungal strains and culture conditions

The WT B05.10 of *B*. *cinerea* was cultured on PDA at 20°C. Gene deletion mutants and the complemented strain were cultured on PDA amended with hygromycin B (Roche, Switzerland) at 100 μg/mL or neomycin G418 (Sigma, USA) at 100 μg/mL.

### Extraction of DNA and RNA, gene cloning, sequences analysis

Genomic DNA extraction and total RNA isolation were carried out with the methods as previously described [45]. Primers for gene cloning were designed with Primer Premier 5.0, multiple sequences alignments were carried out using DNAMAN and the neighbor-joining phylogenetic trees were constructed using MEGA version X programs [46]. *Bcgbl1* DNA and protein sequence were obtained from the National Centre for Biotechnology Information (NCBI) (http://www.ncbi.nlm.nih.gov) and Ensembl Fungi (http://fungi.ensembl.org/Botrytis_cinerea) [25,47].

### Generation of gene disruption and complementation strains

The split-marker homologous recombination method [48] was used to obtain the deletion mutants for the following genes: *Bcgbl1* (*Bcin14g03010*), *BcHsbA1* (*Bcin03g08230*), *BcHsbA2* (*Bcin04g06380*), *BcHsbA3* (*Bcin10g04570*), *Bcmp* (*Bcin12g06300*), and *Bcgpi* (*Bcin04g00460*). The 5’- and 3’-flanking sequences of each gene were PCR amplified from B05.10 genomic DNA as templates with the primers listed in S1 Table, and then fused with the partial hygromycin gene. Two split-market fragments were co-transformed into the protoplasts of the WT strain, and the PEG-mediated transformation [49] was used to obtain transformants. The resultant deletion mutants were first screened on PDA amended with hygromycin B (100 μg/mL) three times, and then verified by PCR and confirmed by Southern blot analysis, using the method described by Tang and colleagues [26]. The primers and probe are listed in S1 Table.

To complement the Δ*Bcgbl1* mutant, the full-length coding sequence of Bcgbl1 and its native promoter were PCR amplified and cloned into *Xba*I/*Pst*I-digested pCAMBIA1300, a binary vector containing the neomycin-resistance gene (*neo*). Then, the complementary vector was transformed into the Δ*Bcgbl1*-30 mutant using the PEG-mediated transformation. The complementation transformants were selected on PDA supplemented with 100 μg/mL neomycin G418 and confirmed by PCR with the primers listed in S1 Table.

### Phenotypic analysis

To analyze growth characteristics, fresh mycelial agar plugs (5 mm-diameter) of each fungal strain were cultured on a PDA plate (9 cm-diameter) at 20°C under darkness. Colony diameter was measured after 2 and 3 days of incubation. The cultures were further incubated for 4 to 30 days for observation of sclerotial development. Conidial germination was assayed using the method described previously by Zhou *et al*. [50]. For the assaying growth characteristics induced by cAMP, exogenous cAMP was added to PDA at final concentrations of 2, 10, and 50 mmol/L. Abiotic stress assays were conducted on PDA amended with 1 mol/L KCl, 1 mol/L NaCl, 0.3 mg/mL Congo Red (CR), or 5 mmol/L H_2_O_2_. The relative mycelial growth rates were calculated according to the colony diameters of each strain at 72 hpi.

### Pathogenicity assays

Leaves of five-week-old tobacco (*Nicotiana benthamiana*) plants were used for plant infection assays. Detached leaves or intact plants were inoculated with 5 mm diameter plugs of 3-day-old cultures of each strain. For macroconidial inoculation, 20 μL of macroconidial suspension (1 × 10^5^ conidia/mL in half strength of PDB) on sterilized filter paper discs (5 mm-diameter) were placed on leaves. For wounding experiments, leaves were wound using a sterilized needle before inoculation, and a mycelial plug or a macroconidial suspension was directly inoculated on the wounded areas. Then inoculated leaves were maintained at 20°C with almost 100% relative humidity. The diameters of lesions were recorded at 72 hpi.

For adhesion assays, 3-day-old mycelial agar plugs (5 mm-diameter) were inoculated on detached tobacco leaves at 20°C with almost 100% relative humidity. After 9 h of inoculation, leaves were gently immersed in water 10 cm in depth for 10 seconds then recorded the number of mycelial plugs that fall off the leaves to calculate the adhesion rate.

### Infection structures observation

Infection-related morphogenesis was observed on the onion epidermis as previously described [51]. Mycelial agar plugs (5 mm-diameter) or droplets of 20 μL of macroconidial suspension (1×10^5^ conidia/mL in half strength of PDB) were deposited onto the hydrophobic side of the epidermis. After 12 h, 24 h, 36 h, and 48 h of incubation in a humid chamber at 20°C, the epidermis was stained with cotton blue before light microscopic evaluation.

Scanning electron microscopy (SEM) was used to observe the formation of infection cushions on tobacco leaves. Mycelial plugs (5 mm-diameter) of strain RoseBc-3 or mutant AT19 were inoculated on tobacco leaves at 20°C. After 12 h, 24h, 72 h, and 120 h of incubation, the leaf tissue from the edge of the inoculation spots was cut into small pieces (2 mm × 2 mm). Leaf specimens for SEM were processed and observed as previously described [51].

### Quantification of the intracellular cAMP content

Two-day-old mycelia were collected from PDB liquid cultures and ground into a powder with liquid nitrogen, then lyophilized for 20 h. The cAMP was extracted as previously described [26]. Intracellular cAMP levels were measured by the Monoclonal Anti-cAMP Antibody Based Direct cAMP ELISA Kit (NewEast Biosciences, Malvern, PA, USA) according to the manufacturer’s protocol.

### Yeast two-hybrid assays

Using the cDNA of the WT strain B05.10 as the template to amplify each tested gene to construct the plasmid for yeast two-hybrid (Y2H) assays, primers used were listed in S1 Table. The coding sequence of each gene was cloned into vectors pGBKT7 and pGADT7, respectively. The pairs of plasmids were co-transformed into *S*. *cerevisiae* strain Y2H Gold (Clontech, USA) following the protocol with positive and negative controls. Transformants were grown at 30°C for 3 days on SD-Leu-Trp medium (synthetic medium without Leu and Trp) and then transferred to SD-His-Leu-Trp medium (synthetic medium without His, Leu and Trp) containing X-α- galactosidase (Clontech, USA).

### Luciferase complementation imaging (LCI) assay

The full-length CDS of *Bcgbl1*, *BCK1*, *BcSte50* and *BcSte11* were constructed into the vectors JW771 (nLUC) and JW772 (cLUC), respectively. The recombinant plasmids were transformed into *A*. *tumefaciens* GV3101. The *A*. *tumefaciens* cells were infiltrated into the leaves of *N*. *benthamiana*. Two days later, luminescence was detected by a CCD camera (Tanon 5100, Shanghai, China) within 5 min after spraying D-luciferin (1 mM) onto the leaves.

### Bmp1 and Bmp3 phosphorylation

Fresh two-day-old mycelia were harvested, finely ground, and suspended in Lysis buffer (50 mM Tris-HCl, pH 7.4, 150 mM NaCl, 1 mM EDTA, 1% Triton X-100) with 1% protease inhibitor cocktail, Phosphatase inhibitor cocktail 2, Phosphatase inhibitor cocktail 3 (Sigma-Aldrich). After homogenization with a vortex shaker, the lysate was centrifuged at 13000 rpm for 20 min at 4°C. The expression and activation of Bmp1 and Bmp3 were detected with the anti-MAPK ERK1/2 antibody (Santa Cruz Biotechnology, USA) and the phospho-p44/42 MAPK antibody (Cell Signaling Technology, USA), respectively. Image J software version 1.54d (National Institutes of Health, Bethesda, MD, USA) was used to quantify the protein bands. The experiment was repeated three times.

### Transcriptome analysis

Mycelia of B05.10 and the Δ*Bcgbl1-*30 mutant growing on PDA and hydrophobic surface (plastic slide) for 24 h were harvested, and immediately frozen in liquid nitrogen and stored at -80°C until RNA extraction. Each type of sample had three independent repeats. Total RNAs of each sample were extracted with RNAiso Plus (TAKARA, 9108). The quality of RNAs was measured by Qubit RNA Assay Kit in Qubit 2.0 Fluorometer, Agilent Bioanalyzer 2100 system at Novogene Bioinformatics Institute (Beijing, China). Library preparation and sequencing were performed at Novogene Bioinformatics Institute. Sequencing libraries were sequenced on an Illumina Hiseq-PE150 platform and the sequencing depth was 6 Gb per library.

### Reverse transcription quantitative PCR analysis

Mycelium samples were harvested to extract total RNA using RNAiso Plus (TAKARA, 9108) and then get the cDNA template with PrimeScrip RT reagent Kit with gDNA Eraser (Perfect Real Time) (TAKARA, RR047A) to detect gene expression level. RT-qPCR was performed with TB Green *Premix Ex Taq* (Tli RNaseH Plus) (TAKARA, RR420A) regent and on a BIORAD CFX96 real-time PCR system following the manufacturer’s instructions. Gene *BcactA* (*Bcin16g02020*) was used as the reference gene and relative quantification was calculated using the 2^-ΔΔCT^ method. The primers used for target genes are listed in S1 Table. This experiment was repeated three times independently, each with three replicates.

### Data analysis

Analysis of variance (ANOVA) in SAS software (SAS Institute, USA, Version 8.0) was used to analyze data on the mycelial growth rate, conidiation, and lesion diameter of strains. Means of each parameter for different strains of *B*. *cinerea* were compared using the Least Significant Difference Test at *P* < 0.05 level.

## Supporting information

S1 Fig Characterization of the T-DNA insertion mutant AT19. (A) Southern blot analysis of the T-DNA insertion event in AT19. Genomic DNA of the WT (RoseBc-3) and AT19 was digested completely with *Sac* I and a partial fragment of the hygromycin resistant cassette gene (*HPT*) was used as the probe. (B) A schematic diagram indicated the position of T-DNA insertion in the promoter region of B0510_5195 in AT19. TF, TR, XLB3, and GUSF, PCR primers. P, probe for Southern blotting. (C) Identification of AT19 by PCR diagnosis with the primer pairs TF/XLB3 and GUSF/TR. (D) Relative transcript levels of *B0510_5194* and *B0510_5195* in WT (RoseBc-3) and AT19. **, significantly different at *P* < 0.01 according to Student’s *T* test.
(TIF)

S2 Fig Deletion and complementation of *Bcgbl1* in *B*. *cinerea*. (A) Schematic diagram showing the strategy to delete *Bcgbl1*. (B) A schematic diagram showing the construct for complementation of *Bcgbl1*. (C) PCR confirmation of deletion of *Bcgbl1* in different mutants. (D) Southern blot confirmation of deletion of *Bcgbl1*. The DNA probe position was shown in A. (E) RT-qPCR confirmation of deletion and complementation of *Bcgbl1* in different mutants.
(TIF)

S3 Fig Exogenous supply of IBMX fails to rescue the defects in mycelial growth of the Δ*Bcgbl1* mutants. The WT and Δ*Bcgbl1* mutants were incubated on PDA supplemented with IBMX (10 μM, 50μM, 1 mM) and DMSO (1/10000, 1/2000, 1/100, v/v) at 20°C in the dark for 3 days.
(TIF)

S4 Fig Bcgbl1 regulates expression of *Bcgas2*. (A) Yeast two-hybrid assay of Bmp1 and BcSte12. (B) RT-qPCR analysis of *Bcgas2* expression during hyphae growth on PDA at 16 hpi in WT (B05.10) and Δ*Bcgbl1*-30.
(TIF)

S5 Fig BcSte12 binding motif in the promoter region of the five Bcgbl1-regulated genes. (TIF)

S6 Fig Effects of deletion of the Bcgbl1-regulated genes on colony morphology and mycelial growth. (A) Colony morphology of different strains on PDA at 20°C in the dark. (B) Mycelial growth rate of different strains. Means ± standard errors labeled with the same letter are not significantly different (*P* > 0.05) according to the least significant difference test.
(TIF)

S7 Fig Phenotypic analysis of deletion mutants of *Bcmp* and *Bcgpi*. (A) Virulence of the WT (B05.10) and the *Bcmp* and *Bcgpi* mutants on tobacco leaves (20°C, 72 h). (B) Lesion size caused by different strains. Means ± standard errors labeled with the same letter are not significantly different (*P* > 0.05) according to the least significant difference test.
(TIF)

S8 Fig Three BcHsbA genes (*BcHsbA1*, *BcHsbA2*, and *BcHsbA3*) are dispensable for osmotic, cell wall, and oxidative stresses adaptation. (A) Sensitivity test of different strains to the osmotic stress (NaCl and KCl), cell wall stress (CR), and oxidative stress (H_2_O_2_). Strains were incubated on PDA plates supplemented with 1 mol/L NaCl, 1 mol/L KCl, 300 μg/mL CR, or 5 mmol/L H_2_O_2_ at 20°C for 3 days. (B) The relative mycelial growth rate of different strains. Means ± standard errors labeled with the same letter are not significantly different (*P* > 0.05) according to the least significant difference test.
(TIF)

S1 Table Primers used in this study. (XLSX)
