# Supplementary figures and images for: The Gβ-like protein Bcgbl1 regulates development and pathogenicity of the gray mold Botrytis cinerea via modulating two MAP kinase signaling pathways
Source: PLoS Pathog. 2023 Dec 4;19(12):e1011839. doi: 10.1371/journal.ppat.1011839 (PMC10721196; doi:10.1371/journal.ppat.1011839)

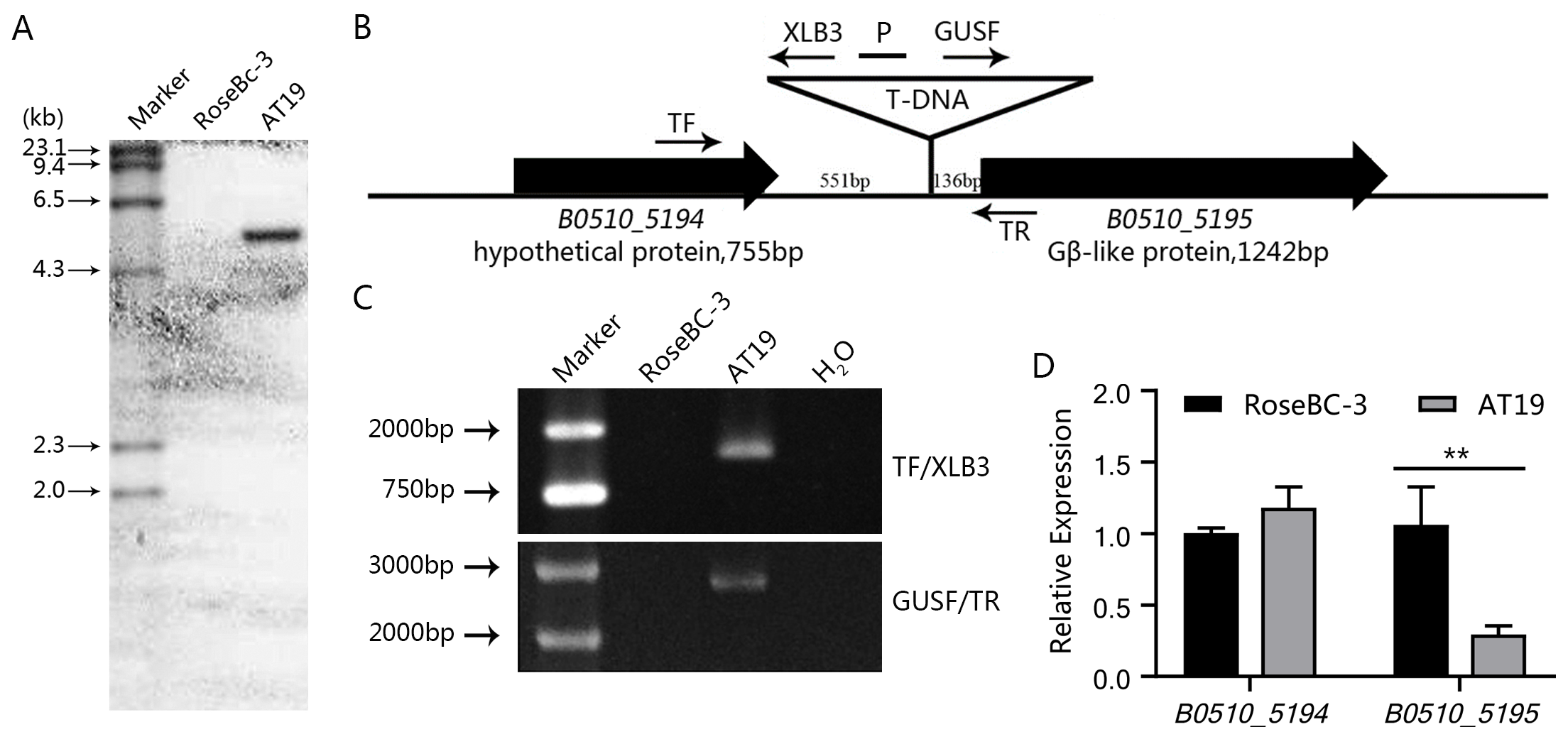

Supplement: S1 Fig — (A) Southern blot analysis of the T-DNA insertion event in AT19. Genomic DNA of the WT (RoseBc-3) and AT19 was digested completely with Sac I and a partial fragment of the hygromycin resistant cassette gene (HPT) was used as the probe. (B) A schematic diagram indicated the position of T-DNA insertion in the promoter region of B0510_5195 in AT19. TF, TR, XLB3, and GUSF, PCR primers. P, probe for Southern blotting. (C) Identification of AT19 by PCR diagnosis with the primer pairs TF/XLB3 and GUSF/TR. (D) Relative transcript levels of B0510_5194 and B0510_5195 in WT (RoseBc-3) and AT19. **, significantly different at P < 0.01 according to Student’s T test. (TIF) [file ppat.1011839.s001.tif]

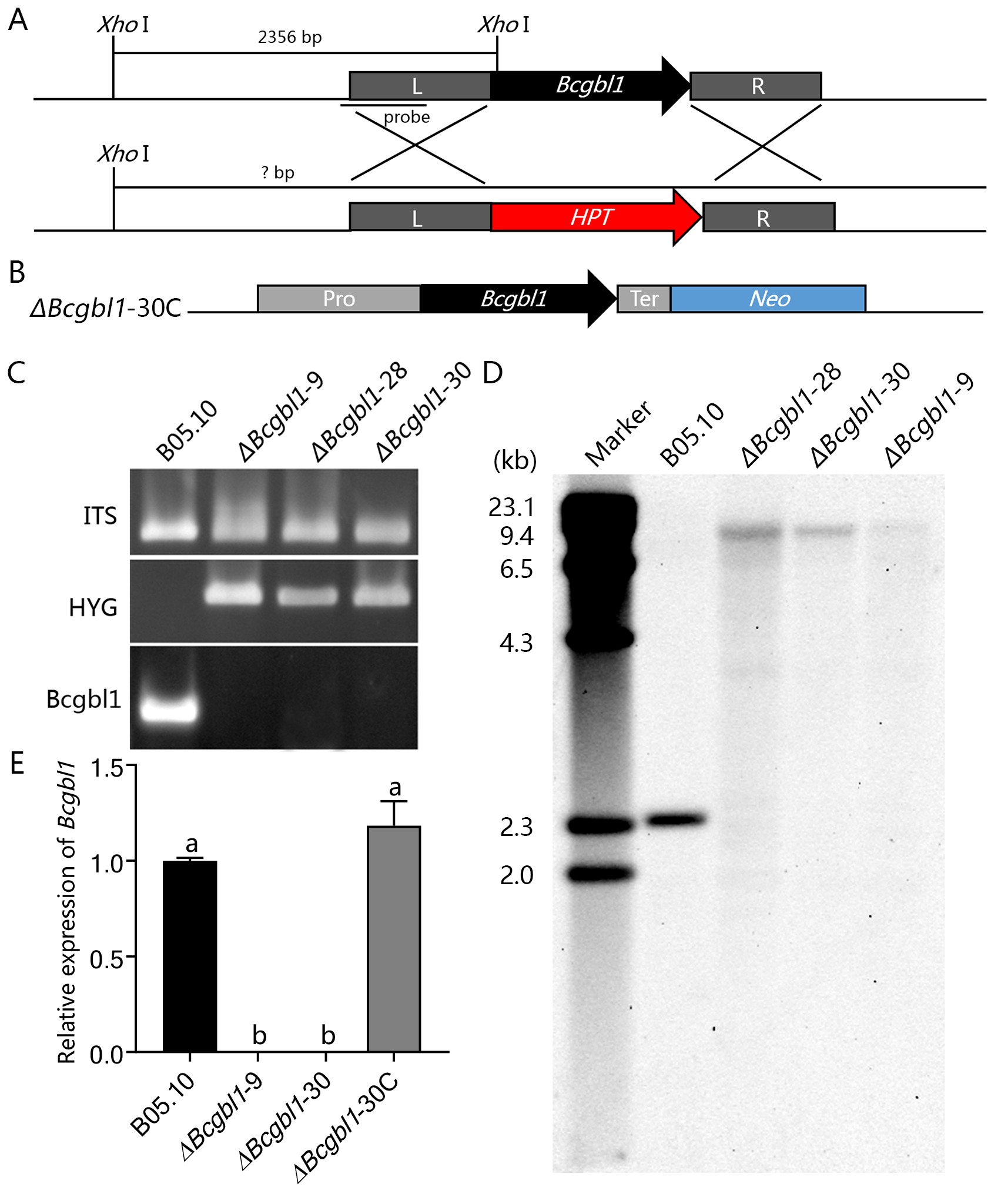

Supplement: S2 Fig — (A) Schematic diagram showing the strategy to delete Bcgbl1. (B) A schematic diagram showing the construct for complementation of Bcgbl1. (C) PCR confirmation of deletion of Bcgbl1 in different mutants. (D) Southern blot confirmation of deletion of Bcgbl1. The DNA probe position was shown in A. (E) RT-qPCR confirmation of deletion and complementation of Bcgbl1 in different mutants. (TIF) [file ppat.1011839.s002.tif]

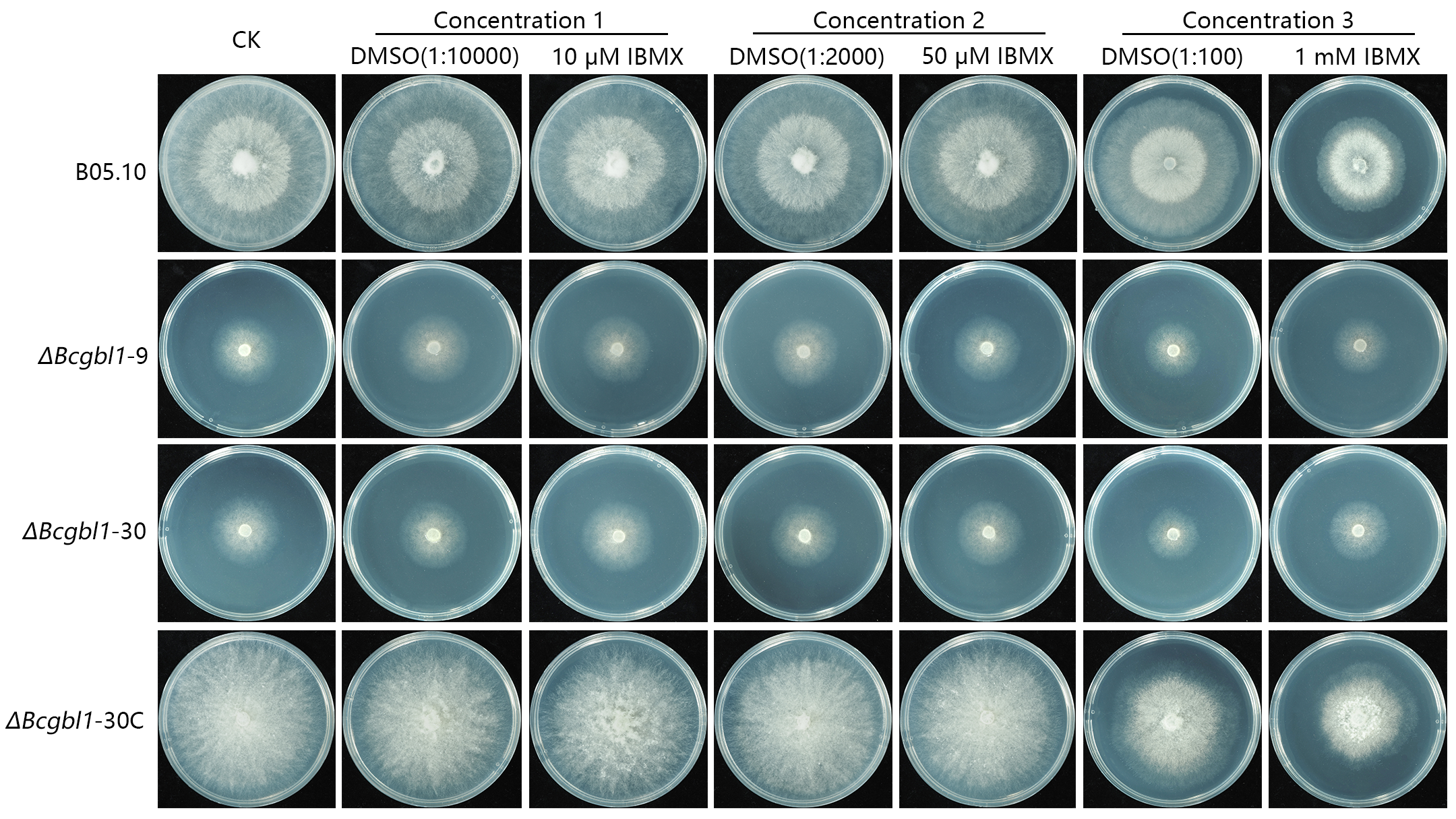

Supplement: S3 Fig — The WT and ΔBcgbl1 mutants were incubated on PDA supplemented with IBMX (10 μM, 50μM, 1 mM) and DMSO (1/10000, 1/2000, 1/100, v/v) at 20°C in the dark for 3 days. (TIF) [file ppat.1011839.s003.tif]

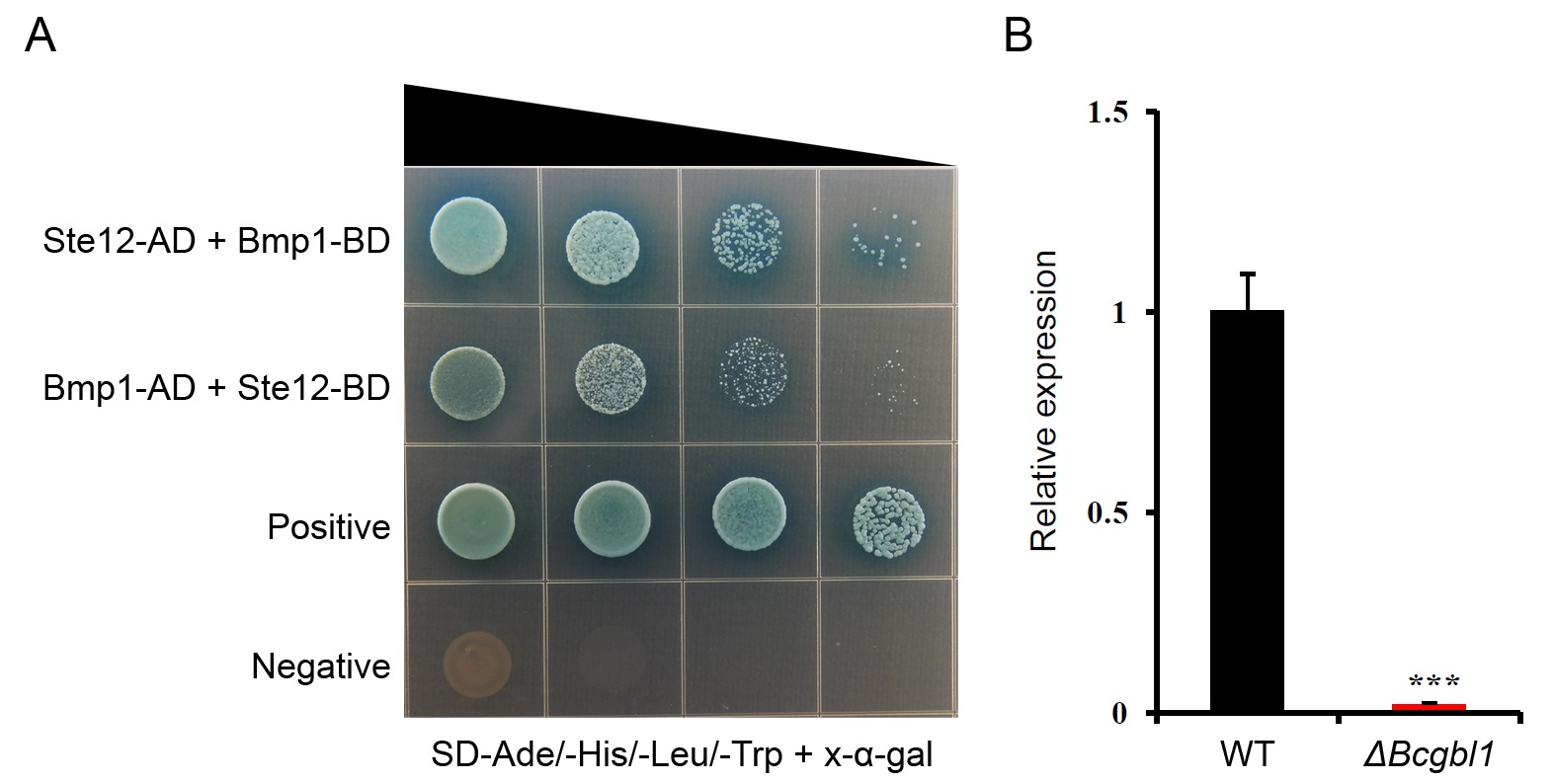

Supplement: S4 Fig — (A) Yeast two-hybrid assay of Bmp1 and BcSte12. (B) RT-qPCR analysis of Bcgas2 expression during hyphae growth on PDA at 16 hpi in WT (B05.10) and ΔBcgbl1-30. (TIF) [file ppat.1011839.s004.tif]

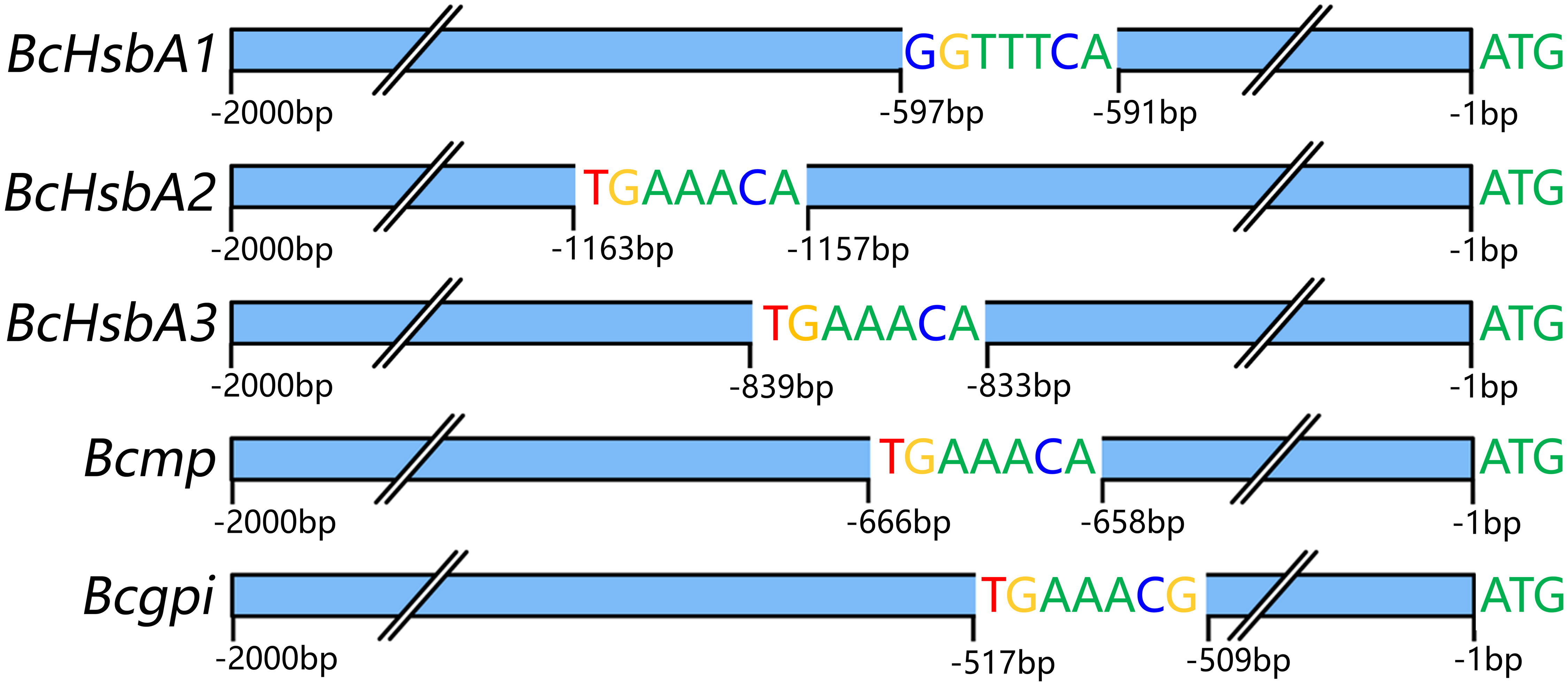

Supplement: S5 Fig — (TIF) [file ppat.1011839.s005.tif]

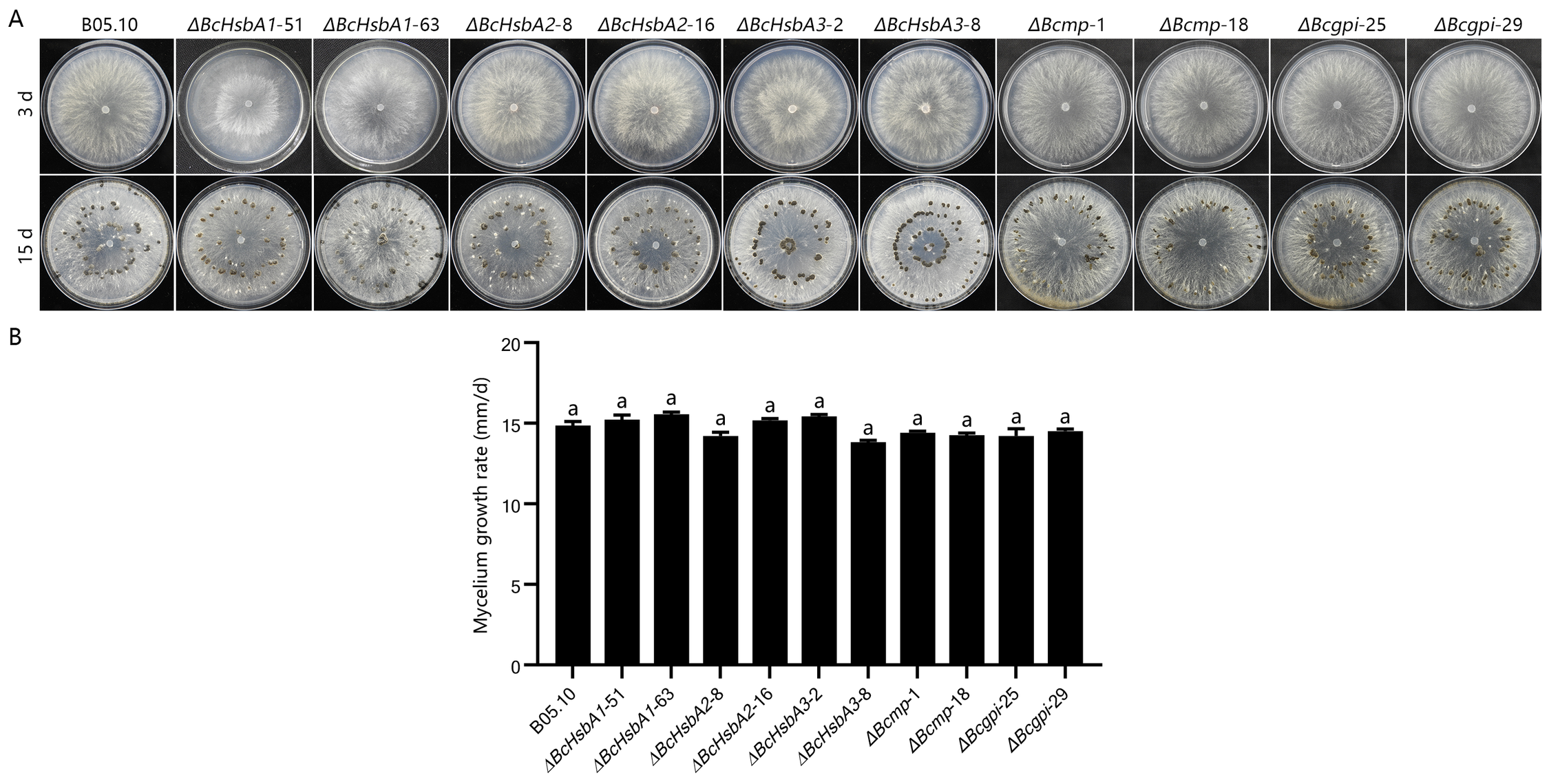

Supplement: S6 Fig — (A) Colony morphology of different strains on PDA at 20°C in the dark. (B) Mycelial growth rate of different strains. Means ± standard errors labeled with the same letter are not significantly different (P > 0.05) according to the least significant difference test. (TIF) [file ppat.1011839.s006.tif]

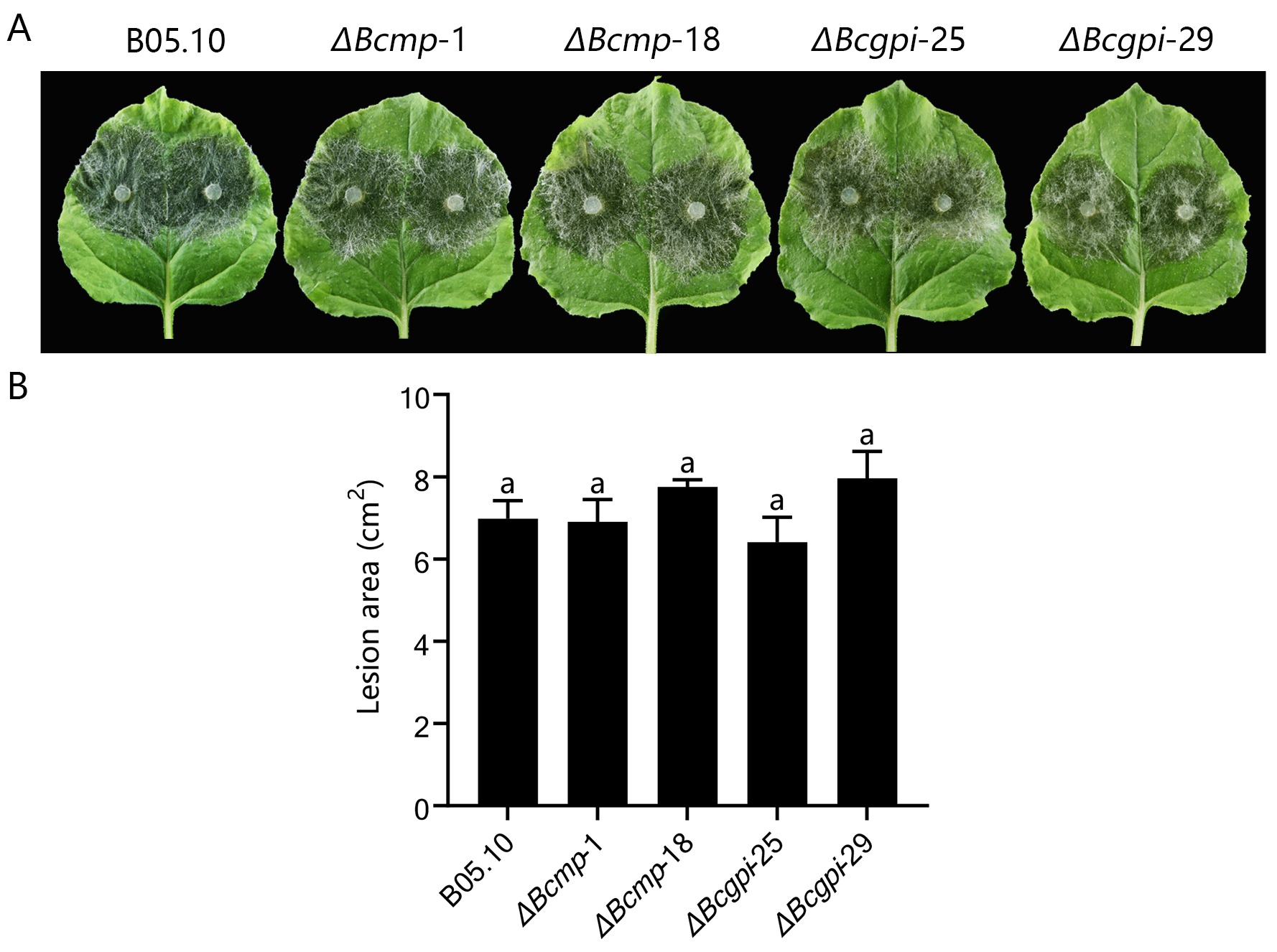

Supplement: S7 Fig — (A) Virulence of the WT (B05.10) and the Bcmp and Bcgpi mutants on tobacco leaves (20°C, 72 h). (B) Lesion size caused by different strains. Means ± standard errors labeled with the same letter are not significantly different (P > 0.05) according to the least significant difference test. (TIF) [file ppat.1011839.s007.tif]

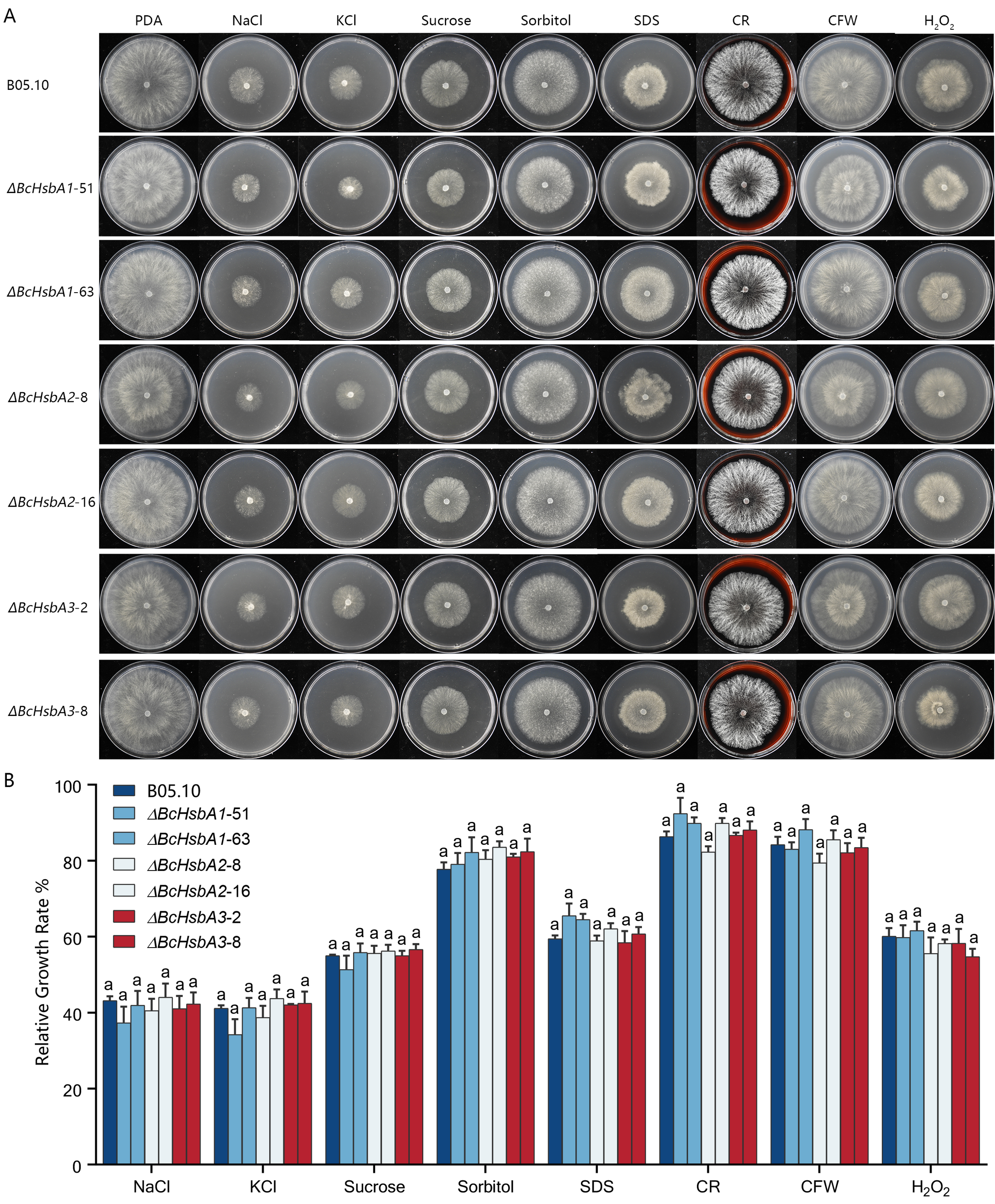

Supplement: S8 Fig — (A) Sensitivity test of different strains to the osmotic stress (NaCl and KCl), cell wall stress (CR), and oxidative stress (H2O2). Strains were incubated on PDA plates supplemented with 1 mol/L NaCl, 1 mol/L KCl, 300 μg/mL CR, or 5 mmol/L H2O2 at 20°C for 3 days. (B) The relative mycelial growth rate of different strains. Means ± standard errors labeled with the same letter are not significantly different (P > 0.05) according to the least significant difference test. (TIF) [file ppat.1011839.s008.tif]
